# Exceptional Thermoelectric Performance of Cu_2_(Zn,Fe,Cd)SnS_4_ Thin Films

**DOI:** 10.1021/acsami.3c17730

**Published:** 2024-02-23

**Authors:** Yu Liu, Paul D. McNaughter, Xiaodong Liu, Andrey V. Kretinin, Jonathan M. Skelton, Feridoon Azough, David J. Lewis, Robert Freer

**Affiliations:** † Department of Materials, University of Manchester, Oxford Road, Manchester M13 9PL, U.K.; ‡ Department of Chemistry, University of Manchester, Oxford Road, Manchester M13 9PL, U.K.; § National Graphene Institute, University of Manchester, Oxford Road, Manchester M13 9PL, U.K.

**Keywords:** thermoelectrics, thin films, Cu_2_ZnSnS_4_ (CZTS), aerosol-assisted chemical vapor deposition (AACVD), Fe and Cd doping, density functional theory

## Abstract

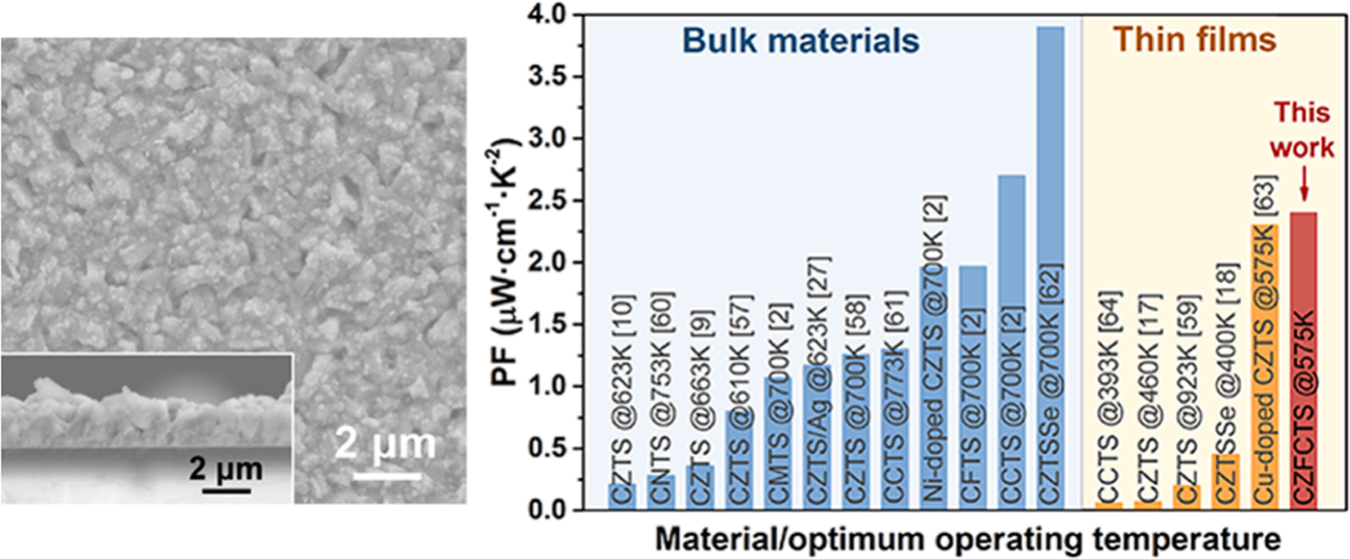

High-quality Cu_2_(Zn,Fe,Cd)SnS_4_ (CZFCTS) thin films based on the parent CZTS were prepared by aerosol-assisted chemical vapor deposition (AACVD). Substitution of Zn by Fe and Cd significantly improved the electrical transport properties, and monophasic CZFCTS thin films exhibited a maximum power factor (PF) of ∼0.22 μW cm^–1^ K^–2^ at 575 K. The quality and performance of the CZFCTS thin films were further improved by postdeposition annealing. CZFCTS thin films annealed for 24 h showed a significantly enhanced maximum PF of ∼2.4 μW cm^–1^ K^–2^ at 575 K. This is higher than all reported values for single-phase quaternary sulfide (Cu_2_BSnS_4_, B = Mn, Fe, Co, Ni) thin films and even exceeds the PF for most polycrystalline bulk materials of these sulfides. Density functional theory (DFT) calculations were performed to understand the impact of Cd and Fe substitution on the electronic properties of CZTS. It was predicted that CZFCTS would have a smaller band gap than CZTS and a higher density of states (DoS) near the Fermi level. The thermal conductivity and thermoelectric figure of merit (*zT*) of the CZFCTS thin films have been evaluated, yielding an estimated maximum *zT* range of 0.18–0.69 at 550 K. The simple processing route and improved thermoelectric performance make CZFCTS thin films extremely promising for thermoelectric energy generation.

## 1 Introduction

With the shortage of fossil fuels and the pressing issue of global warming, there is increasing demand for efficient renewable energy sources. Thermoelectric materials, which convert waste heat to electric power, are attracting increasing attention in this domain. The thermoelectric performance of a material is often described by the thermoelectric figure of merit *zT* (eq 1)
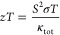
1where *S* is the Seebeck coefficient, σ is the electrical conductivity, *T* is the absolute temperature and κ_tot_ is the total thermal conductivity given by the sum of the electronic thermal conductivity κ_ele_ and the lattice thermal conductivity κ_lat_.^1^ Efficient thermoelectric performance requires a high power factor (PF, *S*^2^ σ) and a low total thermal conductivity, thus maximizing *zT*. Many ternary and quaternary Cu-rich sulfides and specifically quaternary chalcopyrite-like semiconductor compounds of the type I2–II–IV-VI4 (general formula A_2_BCX_4_), derived from zinc blende, exhibit low lattice thermal conductivity due to pronounced structural distortion, and are therefore attractive as potential thermoelectric materials..^2^ Copper zinc tin sulfide, Cu_2_ZnSnS_4_ (CZTS), which belongs to this family of A_2_BCX_4_ compounds, has a band gap of ∼1.5 eV and has been widely studied for photovoltaic (PV) applications.^3^ CZTS normally exhibits three crystal structures:^4^ kesterite (space group *I*4̅), stannite (*I*4̅2*m*), and a primitive mixed CuAu-like (PMCA) structure (*P*4̅2*m*), which are shown in Figure S1. Sphalerite (*F*4̅3*m*) and the disordered kesterite structure (*I*4̅2*m*) have also been reported.^4^ Kesterite is the more thermodynamically stable polymorph, but the energy difference between the kesterite and stannite polymorphs is small,^5^ and transformations between the kesterite and stannite polymorphs have been reported.^3^ To the best of our knowledge, there is no experimental evidence for a transition to the PMCA structure.^6^

Of the large number of studies on CZTS bulk materials and thin films in recent years, most have focused on their preparation methods and properties for solar cell applications,^7,8^ and few have addressed potential thermoelectric applications. Long et al. synthesized bulk CZTS by mechanical alloying (MA) and spark plasma sintering (SPS), and obtained a maximum *zT* of ∼0.22 at 663 K.^9^ Hot-pressed CZTS bulk materials prepared by Sharma et al. exhibited a peak *zT* of ∼0.024 at 623 K.^10^ Due to the similar size of Zn and Cu ions, Cu_Zn_–Zn_Cu_ antisite defects (i.e., Cu–Zn disorder) are present in CZTS at room temperature.^11^ Isotta et al. found that the Cu–Zn disorder in CZTS is temperature-dependent, and demonstrated experimentally, by measurement of the Seebeck coefficients, a transition from relative order (*I*4̅) to disorder (*I*4̅2*m*) around 533 K.^6^ The Cu–Zn disorder leads to an improvement of the Seebeck coefficient without a significant reduction of the electrical conductivity, through modification of the density of states (DoS), thereby enhancing thermoelectric performance.^6,12^ In earlier reports of CZTS-based thermoelectric materials, one of the most common strategies to enhance thermoelectric performance was by ion substitution.^13^ Nagaoka et al. grew CZTS single crystals using a Sn-solvent traveling heater method (THM), and found that the maximum *zT* at 800 K improved from ∼0.05 for stoichiometric CZTS to ∼1.6 for Cu_1.9_ZnSnS_4_ doped with 0.1 mol % Na.^14^ Xiao et al. fabricated polycrystalline CZTS bulk samples by spark plasma sintering (SPS) and found that partial or full substitution of the B-site ions with transition metal atoms such as Ni, Mn, Fe, and Co increased the carrier concentration and thereby improved the electrical conductivity.^2^ Replacing Zn with heavier atoms such as Cd can also strengthen phonon scattering and reduce thermal conductivity.^15^ Jacob et al. prepared Cu_2_Zn_1–*x*_Cd_*x*_SnS_4_ thin films using a sol–gel method and found that both the Seebeck coefficient and electrical conductivity increased with increasing Cd concentration.^16^

However, the modest thermoelectric performance of polycrystalline CZTS limits its exploitation, and, consequently, few investigations have focused on CZTS thin-film thermoelectrics. This is in spite of several attractive advantages of thin films including flexibility, small volume, lightweight, and their potential for use in wearable and other devices. Kumar et al. reported that the electrical transport properties of CZTS thin films deposited by ultrasonically assisted chemical vapor deposition (UACVD) improved with increasing deposition temperature, and CZTS films deposited at 375 °C exhibited the highest PF of ∼7.1 μW K^–2^ m^–1^ at 450 K.^17^ Hsiao et al. prepared a series of Cu_2_ZnSn(S,Se)_4_ (CZTSSe) thin films by spin coating. Samples with a Cu/(Zn + Sn) ratio of 1.01 had the highest PF of 46.52 μW K^–2^ m^–1^ at 400 K.^18^

As a processing technique, aerosol-assisted chemical vapor deposition (AACVD) has the advantages of being low cost and simple to operate, together with easily adjustable atomic ratios compared to many established CVD-type processes.^19^ In this work we employ AACVD to deposit CZTS thin films using metal diethyldithocarbamate complexes as precursors.^20^ In an earlier investigation, Kevin et al. prepared Cu_2_(Zn_*y*_Fe_1–*y*_)SnS_4_ (CZFTS) and Cu_2_(Zn_*y*_Fe_1–*y*_)SnSe_4_ (CZFTSe) thin films using AACVD, and their Fe-rich CZFTS thin films, deposited with 1:1 molar ratio of Fe and Zn diethyldithocarbamate precursors, showed significantly enhanced electrical conductivity.^20^ Inspired by the improvement in electrical conductivity of CZTS thin films by Fe doping, and the potential to increase the Seebeck coefficient and decrease the lattice thermal conductivity by substituting with heavier Cd atoms, we explored the thermoelectric properties of CZTS thin films with partial replacement of Zn by both Cd and Fe to produce Cu_2_(Zn, Fe, Cd)SnS_4_ thin films, referred to hereafter as CZFCTS thin films. We found that the electrical transport properties of CZTS thin films were dramatically improved by Cd and Fe substitution. Density functional theory (DFT) calculations were conducted to understand the effects of Fe and Cd substitution on the electronic structure of CZFCTS. To enhance the thermoelectric properties of CZFCTS thin films, postdeposition annealing was employed to improve film microstructure and increase electrical conductivity. The composition and microstructure of the CZTS and CZFCTS thin films were investigated, and the thermoelectric properties were evaluated. We estimated the maximum *zT* of CZFCTS thin films at 550 K to be in the range 0.18–0.69.

## 2 Methods

### 2.1 Experimental Details

#### 2.1.1 Synthesis and Characterization of Precursors

The molecular precursors [Cu(S_2_CN(C_2_H_5_)_2_)_2_], [Zn(S_2_CN(C_2_H_5_)_2_)_2_], [Fe(S_2_CN(C_2_H_5_)_2_)_3_], [Cd(S_2_CN(C_2_H_5_)_2_)_2_], and [Sn(C_4_H_9_)_2_(S_2_CN(C_2_H_5_)_2_)_2_] were synthesized by reacting sodium diethyldithocarbamate with the corresponding metal salts according to previously reported procedures.^21^ The preparation of the precursors and thermal decomposition analyses are provided in the Supporting Information. C, H, N, and S microanalysis was conducted with a Thermo Flash 2000, and the metal analysis was carried out by inductively coupled plasma atomic emission spectroscopy (ICP-AES) using a Thermo Scientific iCAP 6300 DUO instrument. Thermogravimetric analysis (TGA) and differential scanning calorimetry (DSC) were carried out using a Mettler-Toledo TGA/DSC system from 10–600 °C with a heating rate of 10 °C min^–1^ under nitrogen (N_2_) gas. The TGA and DSC results are presented in Figure S2 and discussed in the Supporting Information.

#### 2.1.2 Deposition of Thin Films by AACVD

CZTS and CZFCTS thin films were deposited on glass substrates by AACVD using stoichiometric ratios of the metal diethyldithiocarbamate precursors. Glass substrates (1.5 × 2.5 cm^2^) were cleaned and sonicated in 20 mL acetone for 30 min, dried using compressed air and placed in sets of 5 (side by side) in a 30 mm diameter glass tube which was loaded into a Carbolite tube furnace. The precursors were dissolved in 10 mL toluene in a two-neck 100 mL round-bottom flask and stirred for 30 min. The flask containing the precursor solution was then attached to the glass tube carrying the substrates, and argon (Ar) carrier gas, with a flow rate of 100 cm^3^ min^–1^, was connected to the other neck of the flask. A Maplin digital ultrasonic humidifier was placed underneath the flask to generate an aerosol of the precursors. The deposition was carried out at 390 °C for 1 h. In this setup, the aerosol droplets were carried by the Ar gas into the hot wall zone of the furnace where evaporation of the solvent and decomposition of the precursor on the substrate occurs, resulting in the deposition of thin films on the surface of the substrates.

For deposition of CZTS thin films, [Cu(S_2_CN(C_2_H_5_)_2_)_2_] (0.36 mmol, 0.13 g), [Zn(S_2_CN(C_2_H_5_)_2_)_2_] (0.18 mmol, 0.065 g) and [Sn(C_4_H_9_)_2_(S_2_CN(C_2_H_5_)_2_)_2_] (0.18 mmol, 0.095 g) were mixed in toluene in a 2:1:1 molar ratio. To enable substitution of Zn by Cd and Fe in the CZFCTS thin films, appropriate amounts of [Zn(S_2_CN(C_2_H_5_)_2_)_2_] were replaced by [Fe(S_2_CN(C_2_H_5_)_2_)_3_] and [Cd(S_2_CN(C_2_H_5_)_2_)_2_], with the precursor ratios in the feed based on earlier work (Table 1).^20^ In total, three CZFCTS compositions were investigated (Table 1). To improve the quality and thermoelectric performance of thin films, postdeposition annealing was carried out at 390 and 470 °C for 1–36 h.

**Table 1:** Deposition Parameters and Precursor Molar Ratios for Preparing CZTS and CZFCTS Thin Films

		molar ratio of precursors	
film	deposition temperature (°C)	Zn/Fe/Cd	Cu/B-site/Sn
CZTS	390	1:0:0	2:1:1
CZFCTS-1	390	1:1:1	2:1:1
CZFCTS-2	390	2:1:2	2:1:1
CZFCTS-3	390	2:1:2	2:1.3:1

#### 2.1.3 Characterization of Thin Films

The structures of the thin films were investigated using grazing-incidence X-ray diffraction (GIXRD) using a PANalytical X’Pert Pro diffractometer with a Cu Kα source (λ = 1.540598 Å) at an incidence angle θ = 3°. The XRD patterns were assigned using the X’Pert Highscore Plus software, and the structures were refined using the Rietveld method with Topas software.^22,23^ The quality of the refinement was determined from the weighted profile *R* values (*R*_wp_) and the goodness of fit (GOF); Rietveld refinements with GOF < 4 and *R*_wp_ < 20% were considered acceptable.^24^ Variable temperature XRD data were collected from room temperature to 300 °C, under vacuum, in a Bruker D8 ADVANCE equipped with an autochanger and an AP TTK450 variable temperature stage.

The microstructure of the thin films was examined using scanning electron microscopy (SEM, Tescan MIRA3 SC) and the elemental compositions were determined using energy-dispersive X-ray spectroscopy (EDX). The size distributions of the grains were determined from the SEM images using the ImageJ software. Transmission electron microscopy (TEM) images and selected-area electron diffraction (SAED) patterns were obtained using a FEI Tecnai 20 TEM operated at 200 kV. High-resolution transmission electron microscopy (HRTEM) images were collected using a FEI Tecnai F30 FEG TEM operated at 300 kV. High-angle annular dark field scanning TEM (HAADF-STEM) images and EDX spectroscopic mapping were performed using a Thermo Fisher Talos F200X FEG TEM operated at 200 kV.

Raman spectra were collected using a Horiba LabRAM HR Evolution spectrometer. Optical absorption spectra of the thin films were recorded using a PerkinElmer Lambda 1050-UV–vis-NIR spectrophotometer and used to estimate the optical band gaps.

The oxidation states of the elements at the surface in the thin films (∼6 nm depth) were determined using X-ray photoelectron spectroscopy (XPS) with a high throughput XPS ESCA2SR spectrometer (Scienta Omicron) with monochromatic Al Kα radiation (*E*_source_ = 1486.69 eV). The XPS data were analyzed with CasaXPS software.

The in-plane electrical transport properties of the thin films were measured using an ULVAC ZEM-3 in a low-pressure helium (He) atmosphere (relative vacuum pressure of ∼−0.09 MPa). The uncertainties in the Seebeck coefficients, electrical conductivities, and power factors were estimated to be 5, 3, and 10%, respectively. Due to the challenges inherent in measuring the thermal conductivity of thin film materials, we concentrated on the samples with the best electrical transport properties. In-plane thermal conductivity measurements of the annealed CZFCTS thin films were carried out using a Linseis thin film analyzer (TFA).^25,26^ The procedures for measuring the thermal conductivity of AACVD-derived thin films using this instrument are detailed in our previous work.^19^ The fragile nature of the membrane on the TFA test chip (on which the films are deposited) makes it very challenging to deposit stress-free films, which do not crack the membrane, and allow the bonding of contact wires for measurements. As the CZFCTS thin films deposited on the TFA test chip were porous, probably leading to an underestimation of thermal conductivity, the data acquired provide the lowest possible values. We therefore also used published thermal conductivity data for CZTS-based bulk samples to define the maximum possible values for comparison.^27^ Further details on the evaluation of the thermal conductivity and *zT* are provided in Supporting Information.

### 2.2 Computational Details

Density functional theory (DFT) calculations were performed with the Quantum ESPRESSO (QE) code.^28,29^ The generalized-gradient approximation (GGA) functional of Perdew, Burke, and Ernzerhof (PBE) was used to approximate the electron exchange and correlation.^30,31^ Ultrasoft pseudopotentials (USPP) were employed to model the ion cores.^32^ A Hubbard *U* value of 5 eV was applied to the d orbitals of the transition metal elements.^30,33,34^ Based on convergence tests for kesterite CZTS, the cutoffs for the Kohn–Sham orbitals (ecutwfc) and charge density (ecutrho) were set to 60 and 600 Ry (1 Ry ≈ 13.606 eV), respectively, and a uniform *k*-point mesh with 4 × 4 × 2 subdivisions was used to integrate the electronic Brillouin zone. A model for Cu_2_Zn_0.25_Fe_0.5_Cd_0.25_SnS_4_ (CZFCTS) was constructed by substituting three Zn atoms with two Fe atoms and one Cd atom in a 2 × 1 × 1 supercell containing 32 atoms. The lattice parameters and atomic positions of both models were relaxed until the force on each atom was less than 10^–3^ Ry, and a total energy tolerance of 10^–6^ Ry was used during the electronic self-consistent field (SCF) cycle. The optimized models were then used to evaluate the electronic density of states (DoS).

## 3 Results and Discussion

### 3.1 Compositional Analysis and Structural Properties

**Figure 1:**
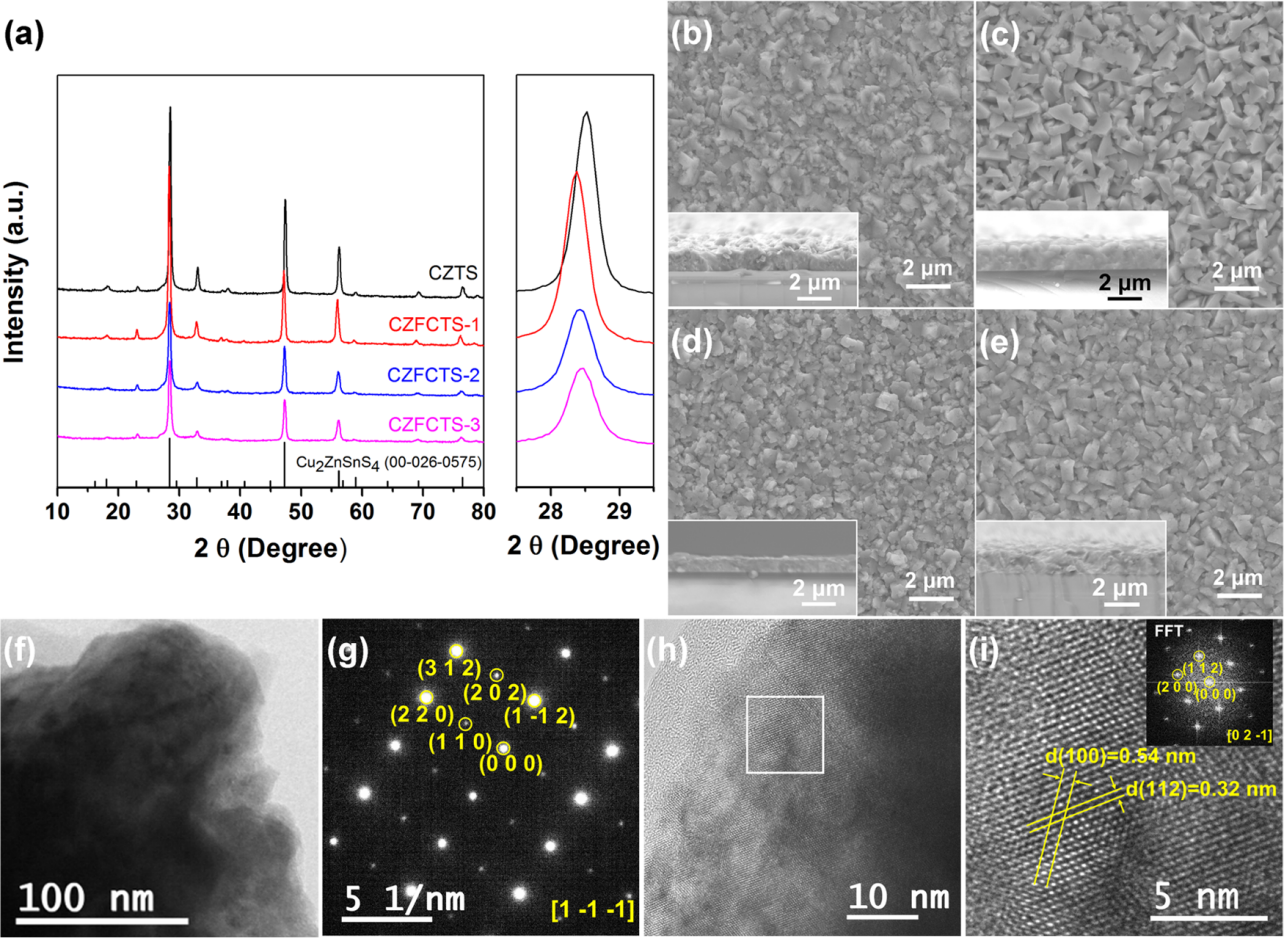
(a) Grazing-incidence X-ray diffraction (GIXRD) patterns for Cu_2_ZnSnS_4_ (CZTS) and Cu_2_(Zn,Fe,Cd)SnS_4_ (CZFCTS) thin films prepared using different molecular precursor ratios. (b–e) Scanning electron microscopy (SEM) images and cross-sectional SEM images of the thin films of (b) CZTS, (c) CZFCTS-1, (d) CZFCTS-2, and (e) CZFCTS-3. Transmission electron microscopy (TEM) images and selected-area electron diffraction (SAED) patterns of the CZFCTS-1 thin film: (f) Low-magnification bright-field TEM image, (g) SAED pattern taken from the entire area shown in (f) but cropped to concentrate on the core details, (h) HRTEM image, and (i) HRTEM image taken from the white region in (h) and FFT image (inset).

Powder XRD patterns for the CZTS and CZFCTS thin films are presented in Figure 1a. The XRD patterns of all the thin films correspond approximately to tetragonal CZTS (JCPDS: 00–026–0575). The major XRD peak at ∼28.5° exhibits a slight shoulder on the left side, which might be due to impurities, such as Cu–S binaries. However, the shoulder was too weak to be refined reliably by Rietveld techniques. It is difficult to distinguish between kesterite and stannite using XRD due to the similarity of the atomic structures.^5^ Here, the kesterite structure is adopted for structural analysis, and further details of the CZTS structure are provided in Section 3.2 below. For the CZFCTS thin films, the (112) reflection at ∼28.5° shows a clear shift to lower angles, indicating lattice expansion. This can be explained by the radius of four-coordinate Cd^2+^ (0.78 Å) being much larger than that of four-coordinate Zn^2+^ (0.60 Å).^35^ The XRD data were refined using the Rietveld method; the refined XRD patterns are presented in Figure S6 and the refined lattice parameters are presented in Table 2. The results are discussed below.

**Table 2:** Lattice Parameters of the Thin Films Prepared in this Study from Rietveld Refinement of X-ray Diffraction (XRD) Data

	lattice parameters (Å)					
film	*a*	*c*	δ	cell volume (Å^3^)	*R*_wp_	GOF
CZTS	5.4272(6)	10.852(2)	0.02	319.65(9)	3.91	1.74
CZFCTS-1	5.4529(4)	10.887(4)	0.17	323.70(14)	4.49	1.93
CZFCTS-2	5.4486(8)	10.888(4)	0.08	323.24(15)	4.34	1.76
CZFCTS-3	5.4438(15)	10.870(3)	0.16	322.1(2)	5.29	1.86

On the basis of in situ variable temperature XRD analysis of thin films during heating and cooling cycles from room temperature to 300 °C (data for CZFCTS-1 is shown in Figure S3), the thin films are thermally stable with no evidence of the formation of secondary phases. The small shift in all the reflections to lower angles during heating is attributed to thermal expansion of the lattice which was found to contract on cooling.

The relationship between the mole fractions of the metal atoms in the AACVD feed and the stoichiometry found in the thin films is shown in Figure S4. A close-to-stoichiometric CZTS thin film can be prepared using AACVD. The CZFCTS thin films have similar ratios of Cu:Sn:B-site (difference below 6%) (Figure S4a), and the metal contents of the CZFCTS thin films are consistent and correspond well with the mole fractions of the metal precursors in the AACVD feed (Figure S4c). The stoichiometry of the CZFCTS thin films can thus be controlled to some degree by adjusting the precursor ratios. We note that the CZFCTS-1 thin films contain the highest Fe content, which, as discussed below, results in the most favorable electrical properties.

The results from Rietveld refinement^22^ of the XRD data are presented in Table 2. The GOF values are below 2 and the *R*_wp_ are less than 10%, indicating acceptable refinements.^24^ For an ideal tetragonal CZTS structure, the ratio *c*/2*a* should be equal to 1. Deviations from this ideal value, defined as δ = (1 – *c*/2*a*) × 100% express the degree of tetragonal distortion.^36^ As shown in Table 2, all CZFCTS thin films have larger *a* and *c* lattice parameters and cell volumes than CZTS thin films confirming lattice expansion and successful substitution of Cd and Fe at the Zn sites in the CZTS lattice. Moreover, the δ values for the CZFCTS thin films are much higher than for the CZTS films, indicating more distorted unit cells. The CZFCTS-1 thin films exhibit both the largest cell volume and the largest δ parameter, indicating the greatest lattice expansion and the highest degree of distortion, as a result of the larger Cd and Fe content and reduced Zn content.

Representative surface morphologies of the CZTS and CZFCTS thin films from SEM characterization are shown in Figure 1b–e. The CZTS and CZFCTS-2 films are the thinnest at around 1.5 μm and consist of irregularly shaped crystallites with grain sizes of about 0.6 μm. In contrast, the CZFCTS-1 and CZFCTS-3 films are thicker (∼2 μm) and show stacked thick, flake-like crystallites with a larger grain size of ∼0.8 μm. The morphologies of both the CZTS and CZFCTS thin films are similar to those of the sulfide and selenide films reported by Kevin et al.^20^ and Li et al.^37^ The differences in grain size and thickness of the present CZFCTS thin films could be related to the compositional differences. Although we do not have sufficient data to propose or confirm a growth mechanism, Tanaka et al. previously noted that the ratio of Cu/(Zn+Sn) can affect grain size in CZTS thin films.^38^

The nanostructure of the CZFCTS-1 thin films was further investigated by TEM analysis. Figure 1f,g shows a TEM image and the corresponding SAED pattern along the [11̅1̅] zone axis of the CZFCTS-1 film. The SAED pattern shows well-defined reflections indicative of high crystallinity, which are similar to those reported by Sui et al.^39^Figure 1h,i shows the HRTEM image and corresponding FFT pattern along the [021̅] zone axis. The *d*-spacing of the (100) plane in Figure 1i is ∼0.54 nm, which corresponds to the refined lattice parameter in Table 2. The *d*-spacings of the (112) and (110) planes are ∼0.32 and ∼0.38 nm respectively, in good agreement with the work of Digraskar et al.^40,41^ HAADF-STEM analysis and EDX elemental maps for a CZFCTS-1 thin film (Figure S5) confirm that Cu, Zn, Fe, Cd, Sn, and S are uniformly distributed, providing further evidence for the successful incorporation of Cd and Fe into the CZTS lattice.

### 3.2 Raman, UV–Vis Absorption, and X-ray Photoelectron Spectroscopies

The Raman spectrum of the CZTS thin film (Figure S7a) shows an intense peak at 332 cm^–1^ and shoulders at 286 and 367 cm^–1^, consistent with earlier investigations on kesterite CZTS films.^3^ There are no secondary impurity peaks in the spectra of the CZTS and CZFCTS thin films. Compared to CZTS, the features in the spectra of the CZFCTS thin films are shifted to lower wavenumbers and the intensity of the main peak decreases; the latter might be due to internal strain and local structural inhomogeneities introduced by the high levels of Fe and Cd doping.^42,43^ These changes could also be related to the disorder that develops at the structural transition from the kesterite to the stannite polymorph. Similar changes in the Raman spectra were reported for Cd-doped CZTS thin films by Zhang et al.^44^ and for Cu_2_(Zn,Fe)SnS_4_ thin films by Khadka and Kim.^45^

The optical band gap energies of the thin films were estimated; the procedures and data are shown in the Supporting Information (Figure S7b). The band gap of the CZTS thin films was determined to be ∼1.7 eV, agreeing well with documented values for CZTS thin films.^3,46^ The band gaps of the CZFCTS thin films range from 1.3 to 1.6 eV, with the band gap of CZFCTS-1 (which has the largest Fe content) being the smallest. The band gaps of the CZFCTS films are 0.1–0.4 eV smaller than those of the CZTS films, consistent with earlier work.^3,47^ The reduction of band gap could arise from the weakened antibonding component of s-s and s-p hybridization between Sn and S leading to a reduced conduction band minimum (CBM).^3,47^

**Figure 2:**
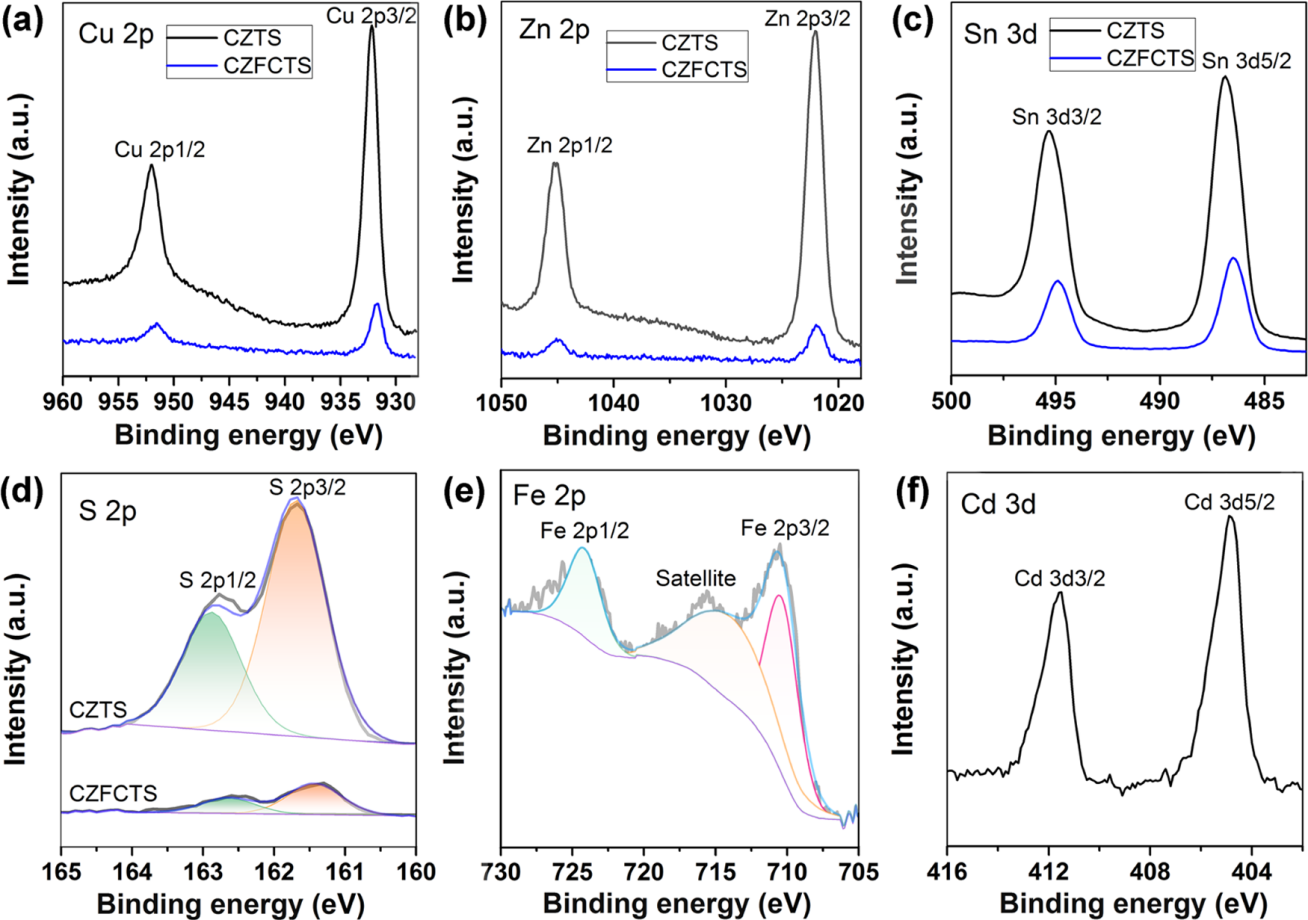
High-resolution X-ray photoelectron spectroscopy (XPS) measurements of the Cu 2p (a), Zn 2p (b), Sn 3d (c), and S 2p (d) binding energies in CZTS and CZFCTS-1 thin films, together with the Fe 2p (e) and Cd 3d (f) binding energies in CZFCTS-1 thin films.

Figure 2 shows high-resolution XPS measurements on the CZTS and CZFCTS-1 thin films. From these, the binding energies and the oxidation states of the elements were determined (Table S1). For the CZTS films, the two peaks located at 952.1 and 932.2 eV are attributed to the Cu 2p_1/2_ and Cu 2p_3/2_ energy levels of Cu(I) (Figure 1a); the peaks at 1045.2 and 1022.1 eV to the Zn 2p_1/2_ and Zn 2p_3/2_ states of Zn(II) (Figure 2b); the peaks at 495.3 and 486.8 eV to the 3d_3/2_ and 3d_5/2_ states of Sn(IV) (Figure 2c);^48^ and the peaks at 162.8 and 161.7 eV to the S 2p_1/2_ and S 2p_3/2_ of S^2–^ (Figure 2d). Compared to the XPS measurements on the CZTS thin films, the peaks for Cu, Zn, Sn, and S in the spectra of the CZFCTS-1 films are displaced slightly to lower binding energies, suggesting a decrease in the oxidation states and reduced surface oxidation.^49^ There is however almost no change in the peak separations after Cd and Fe doping. The peaks for Fe 2p_1/2_ and Fe 2p_3/2_ are located at 724.5 and 711.0 eV (Figure 2e), which is indicative of Fe(II), and similar to previous measurements on Cu_2_FeSnS_4_ and Cu_2_Zn_1–*x*_Fe_*x*_SnS_4_ thin films.^50,51^ The Cd 3d_3/2_ and Cd 3d_5/2_ peaks are located at 411.6 and 404.9 eV (Figure 2f) and can be attributed to Cd(II). The shifts of the Cu, Zn, Sn, and S spectral features and the presence of Fe and Cd peaks in the XPS spectra for the CZFCTS thin films provide further evidence for the successful introduction of Fe and Cd into the CZTS lattice.^52^

### 3.3 Electrical Transport Properties

**Figure 3:**
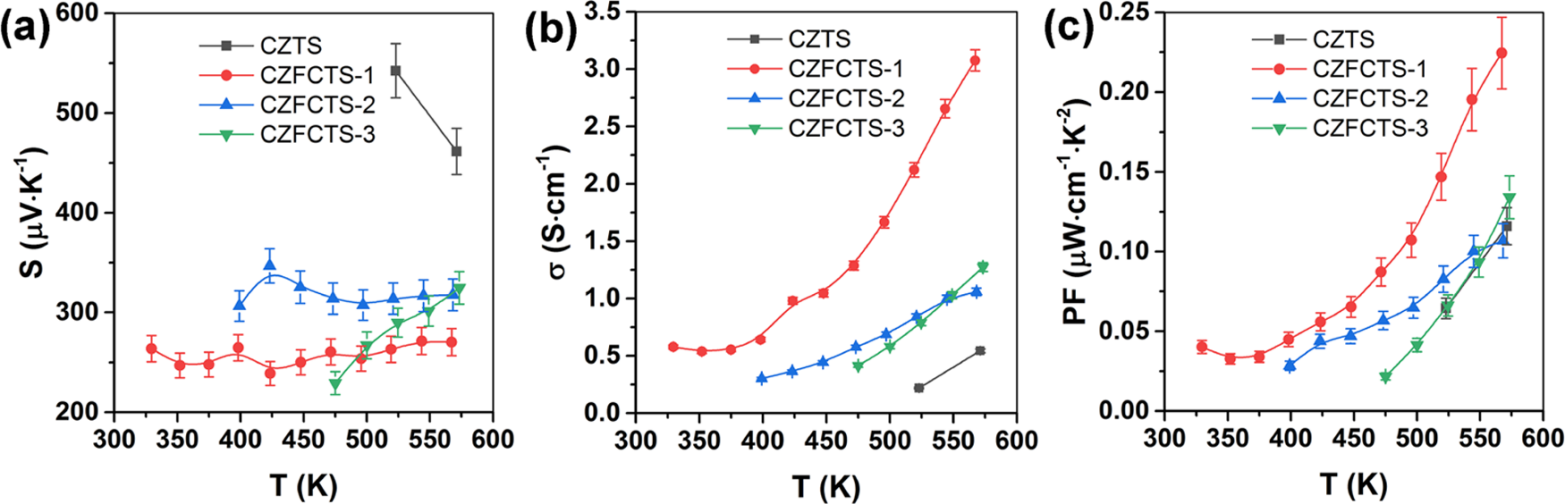
Temperature-dependent Seebeck coefficient *S* (a), electrical conductivity σ (b), and power factor PF (c) of the CZTS and CZFCTS thin films. The uncertainty bars indicate uncertainties of 5, 3, and 10% in the measured *S*, σ, and PF, respectively.

The temperature-dependent electrical transport properties, viz. the Seebeck coefficient *S*, electrical conductivity σ, and power factor PF of the CZTS and CZFCTS thin films are shown in Figure 3. The positive *S* (Figure 3a) suggests p-type behavior for all the thin films. In all cases the *S* and σ increase with increasing temperature, consistent with the findings of previous studies.^2^ A particularly high *S* of ∼540 μV K^–1^ is obtained for the CZTS thin films, but below 525 K the σ values are low, ≤ 0.2 S cm^–1^, and are too low to be reliably determined. In contrast, the CZFCTS thin films exhibit the more traditional inverse relationship between the Seebeck coefficient and electrical conductivity, with lower *S* but higher σ (Figure 3b). The CZFCTS-1 films exhibit the highest σ, probably as a result of increased carrier concentration, through the substitution of Zn with Fe and Cd, together with the larger grain size. The CZFCTS-2 and CZFCTS-3 thin films exhibit similar σ, higher than that of CZTS, but the CZFCTS-2 films have larger *S*, suggesting that the higher σ of the CZFCTS-2 thin films arises partially from improved carrier mobility. The balance of these parameters favors the CZFCTS-1 films, which have the highest overall PF of ∼0.22 μW cm^–1^ K^–2^ at 575 K (Figure 3c). Multiple ZEM measurements for the CZFCTS-1 thin film (Figure S8) confirm the repeatability of the data.

### 3.4 DFT Calculations of the Electronic Properties of CZTS and CZFCTS

**Figure 4:**
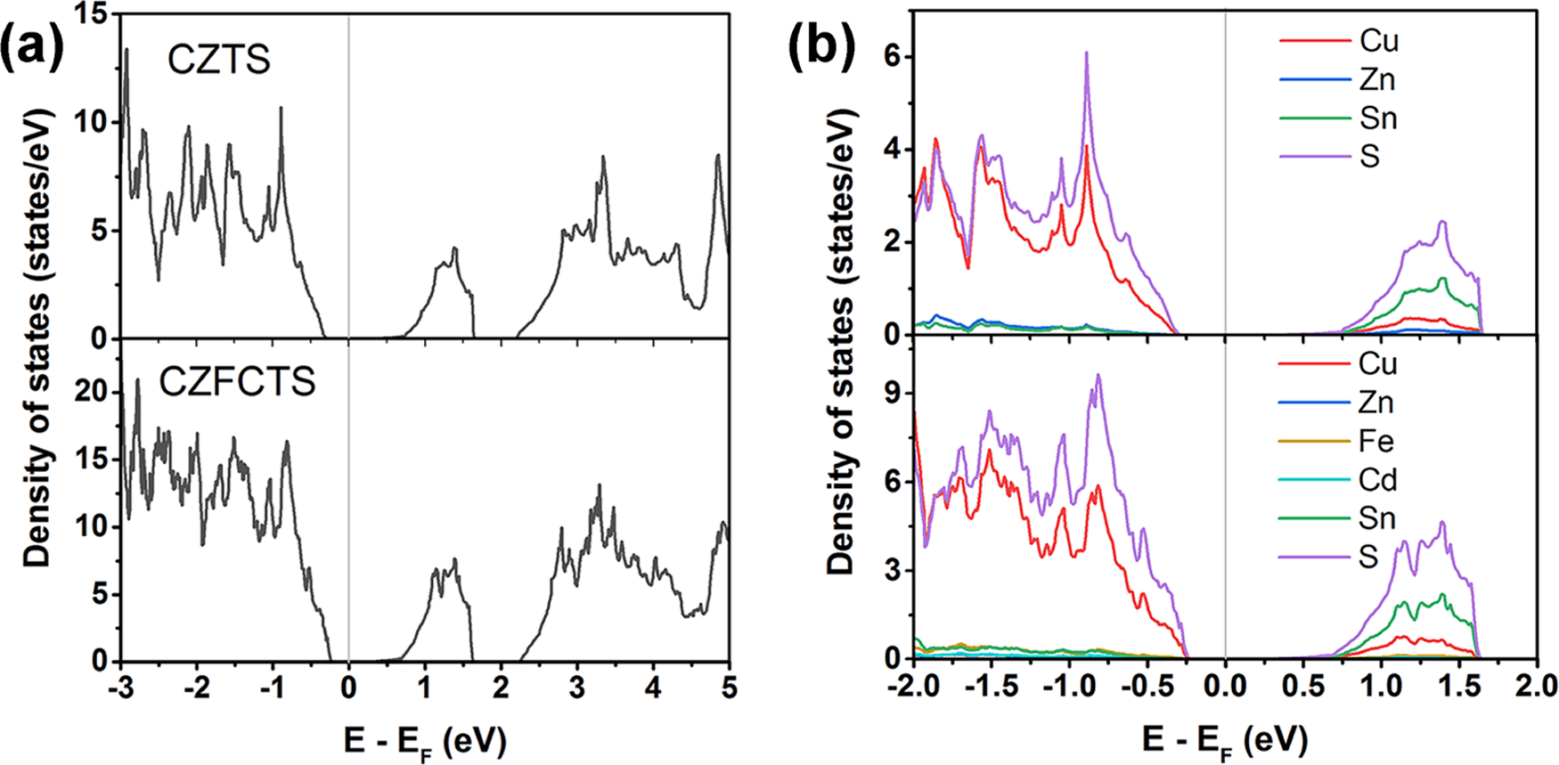
Calculated total electronic density of states (TDoS); (a) and atom-projected (partial) density of states (PDoS; (b)) for CZTS and a model of CZFCTS.

To help understand the effects of Cd and Fe substitution on the electronic properties of CZTS, electronic-structure calculations were performed on CZTS and a model of Cu_2_Zn_0.25_Fe_0.5_Cd_0.25_SnS_4_. The optimized lattice parameters of CZTS were *a* = 5.49 and *c* = 10.95 Å, close to the values for bulk CZTS from simulations and experiments.^14^ The calculated total and partial density of states (TDoS/PDoS) of the two models are compared in Figure 4. The Fermi level is close to the valence band, indicating predominant p-type conductivity as suggested by our Seebeck measurements. The calculated band gap of CZTS is ∼1.0 eV, which is similar to previous computational studies^34^ but smaller than the experimental value, most likely due to the well-known tendency of GGA functionals to underestimate band gaps.^53^ The calculated band gap for the CZFCTS model is ∼0.8 eV, and the reduction compared to CZTS is in the same range as the change in optical band gaps obtained from our measurements. A possible reason for the reduced optical band gap (Figure S7b) is given above. From the calculated DoS, the valence-band maximum (VBM) of CZFCTS also lies closer to the Fermi level, suggesting that Cd and Fe doping might form an acceptor level above the CZTS VBM and lead to an increase in the hole carrier concentration. Cd and Fe substitution also increases the density of states (DoS) at the top of the valence band near the Fermi level, which may improve the Seebeck coefficient.^54^ The PDoS data suggests that Cd/Fe do not contribute states at the VBM; the increase in the DoS in the doped model is therefore due to a shift in the energy of the Cu/S states to lower binding energies. This is consistent with the change in oxidation, which is evident from the XPS data. These synergistic effects may allow the CZFCTS films to circumvent the usual trade-off between the Seebeck coefficient and electrical conductivity, i.e., supporting an enhanced electrical conductivity without a large degradation of the Seebeck coefficient.

### 3.5 Enhancement of Thermoelectric Properties by Postdeposition Annealing

**Figure 5:**
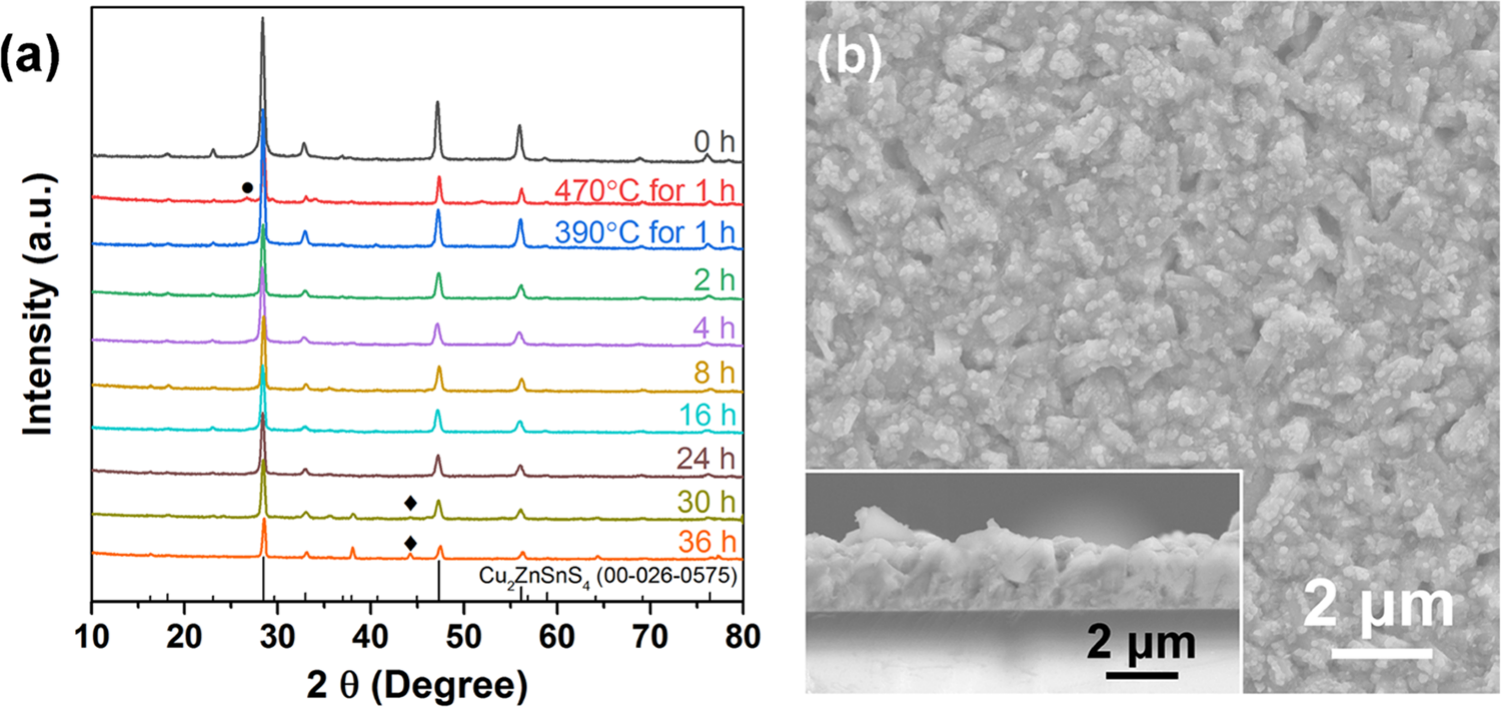
(a) XRD patterns for as-prepared CZCFTS-1 thin films (0 h) and films annealed for up to 36 h. Impurity peaks assigned to Cu_2_S and FeS are marked by circles and diamonds, respectively. (b) SEM and cross-sectional images of a CZFCTS-1 thin film annealed at 390 °C for 24 h.

In order to improve the quality of the CZFCTS-1 thin films and, in particular, to attempt to enhance thermoelectric performance, the CZFCTS-1 thin films were annealed at 390 and 470 °C for 1 h after deposition. As shown in the XRD patterns (Figure 5a), a Cu_2_S secondary phase developed in the CZFCTS-1 film annealed at 470 °C for 1 h, but no secondary phase formation was observed in the film annealed at the lower temperature. Consequently, an annealing temperature of 390 °C was adopted to investigate the effect of different annealing times. The XRD patterns (Figure 5a) show that CZFCTS-1 films annealed at 390 °C for 1–24 h were all monophasic, with no evidence of impurity peaks. In films annealed for longer than 24 h, however, a secondary peak was observed and assigned to FeS based on the appearance of an associated Raman peak at 220 cm^–1^ from the FeS asymmetric stretching mode (Figure S9).^55^ With increasing annealing time, the XRD peaks become slightly weaker and broader, suggesting a limited reduction in the degree of crystallinity.

Plan view and cross-sectional SEM images of CZFCTS-1 thin films annealed at 390 °C for different times are shown in Figures 5b and S10–S12. The microstructures of the films are dominated by thick, flake-like grains. With increasing annealing time, the morphology of the films becomes denser and more compact, with less evidence of pinholes. The sizes of grains, more specifically lengths, within the central 60% of the SEM images, were determined using ImageJ software, and the size distributions are shown in Figure S13. As the annealing time increased from 0–8 h, the average grain length increased from ∼0.81 to ∼1.33 μm, implying a reduction in scattering by grain boundaries. However, further increase in the annealing time to 36 h had little impact, with the grain sizes remaining around 1.30 μm. EDX data for the samples annealed for 36 h confirmed the presence of the FeS secondary phase (Figure S12).

**Figure 6:**
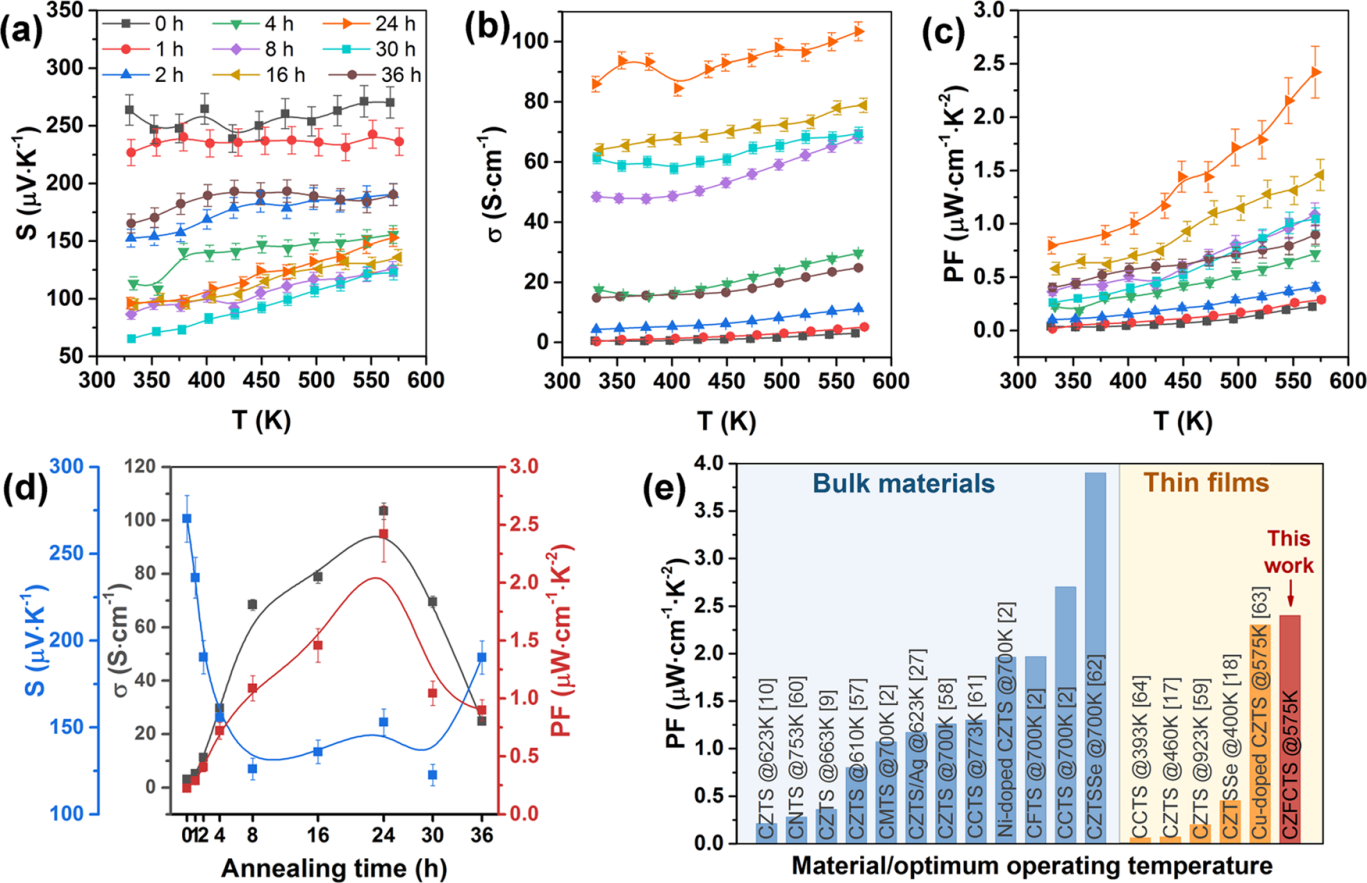
Temperature dependence of (a) the Seebeck coefficients (*S*), (b) electrical conductivity (σ), and (c) power factor (PF) for as-prepared (0 h) and annealed CZFCTS-1 thin films. (d) Dependence of *S*, σ, and PF at 575 K on postdeposition annealing time. (e) Comparison of the maximum PF value obtained in this work and published data for single-phase polycrystalline quaternary sulfide (Cu_2_BSnS_4_ (CBTS); B = Mn, Fe, Co, Ni) bulk and thin film materials.^2,9,10,17,18,27,57−64^ The uncertainty bars show uncertainties of 5, 3, and 10% in the *S*, σ, and PF values, respectively.

The charge transport properties of the CZFCTS-1 thin films, both before and after annealing, are compared in Figure 6a–c (data for repeat measurements are shown in Figure S14). In all cases, the Seebeck coefficient, electrical conductivity, and power factor increase with temperature but show a complex dependence on the annealing time. In order to explore this further, properties measured at 575 K are plotted as a function of annealing time in Figure 6d. For the CZFCTS thin films annealed for 0–8 h, there is a gradual reduction of *S* and an increase in σ with increasing annealing time. The inverse relationship between *S* and σ implies that the change in σ is due to changes in the carrier concentration. This correlates with the fact that after annealing for 8 h, the microstructure is denser and more compact, the grain size reaches its maximum value (Figures S10–S13) and the limited Fe and Cu migration to the surface could contribute to an increase of carrier concentration.^56^ On further increase of the annealing time from 8–24 h, σ steadily increases and *S* increases slightly (Figure 6d), reflecting improved microstructure and enhanced carrier mobility. For the longest annealing times of 30 and 36 h, there is a rapid decrease in σ and an increase in *S*. This may have been caused by a decrease in carrier concentration related to the reduction of Fe in the CZFCTS matrix induced by the segregation of FeS (Figure S12), and electron scattering caused by the presence of the secondary phase (c.f. Figure 5a). During annealing, cationic disordering, such as Cu–Zn disorder mentioned in the introduction might have increased;^2^ however, it is very challenging to confirm this experimentally. The highest σ of ∼102 S cm^–1^ was obtained at 575 K for the CZFCTS-1 thin film annealed for 24 h.

The PF of the CZFCTS-1 thin films was substantially improved by annealing (Figure 6c). Compared to the CZTS films (Figure 3), the CZFCTS-1 thin films annealed for 24 h show significant enhancement in maximum PF, by a factor of ∼22, with a PF of ∼2.4 μW cm^–1^ K^–2^ at 575 K. Figure 6e provides a comparison of the maximum PF values obtained in this work to that of published data for analogous polycrystalline quaternary sulfide (Cu_2_BSnS_4_ (CBTS), where B = Mn, Fe, Co, Ni) bulk materials and thin films.^2,9,10,17,18,27,57−64^ Our maximum PF values are significantly higher than all previous reports for single-phase CZTS-based thin films and most polycrystalline bulk materials. This outstanding result demonstrates that cosubstitution of high levels of Cd and Fe into CZTS is an effective approach to increasing the power factor of thin films, and, furthermore, that postdeposition annealing can both enhance film quality and significantly improve charge transport.

Accurate determination of *zT* for thin films is much more difficult than for bulk materials, as the measurement of the in-plane thermal conductivity of thin films is a considerable engineering challenge. To the best of our knowledge, the thermal conductivity and *zT* values for CZTS-based thin films have not previously been reported. In the present work, the total thermal conductivity κ_tot_ of the CZFCTS-1 thin films was determined by TFA. The electronic thermal conductivity κ_ele_ was calculated from the electrical transport measurements using the Wiedemann–Franz Law as (eq 2)^65^
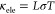
2where the Lorenz number *L* was estimated using eq 3.^66^
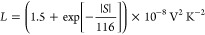
3

The calculated *L* and κ_ele_ are shown in Figures S15b and 7a, respectively. This data allows the lattice thermal conductivity κ_lat_ to be determined as κ_lat_ = κ_tot_ – κ_ele_.

**Figure 7:**
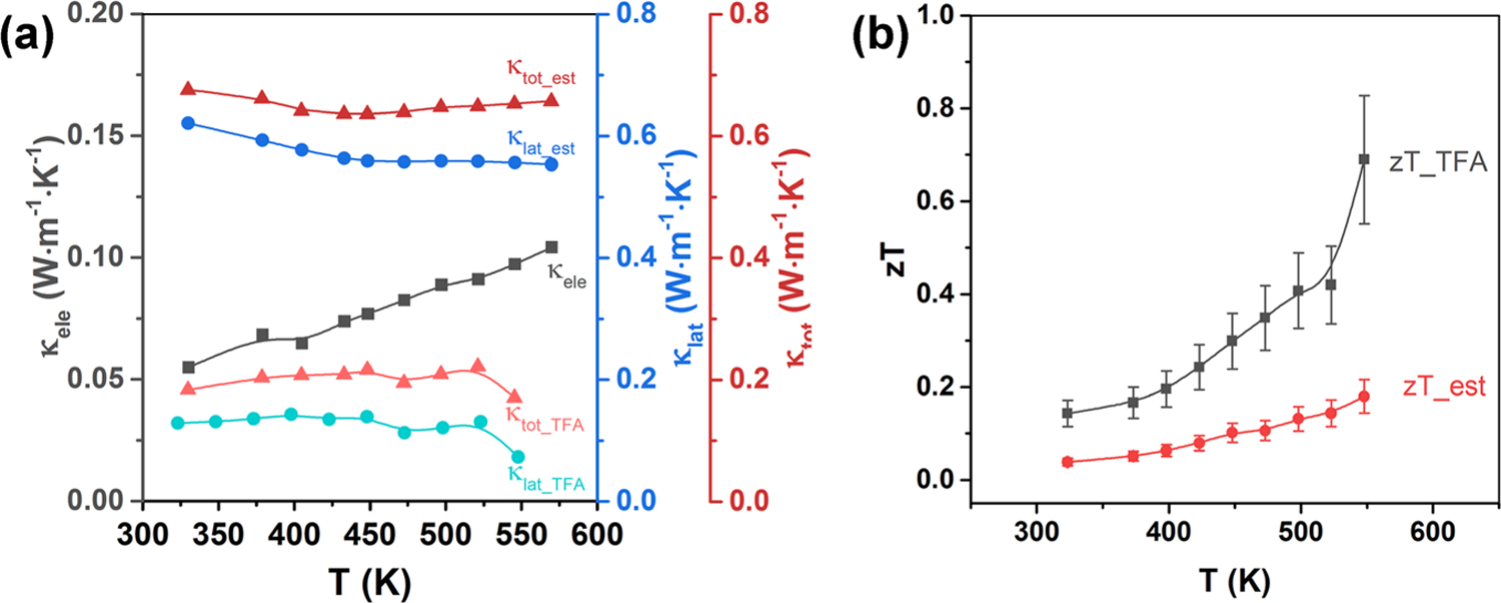
(a) Temperature dependence of the total thermal conductivity κ_tot_ and the electronic and lattice components κ_ele_/κ_lat_ for our 24 h annealed CZFCTS-1 thin film. As described in the text, the κ_tot_TFA_ and κ_lat_TFA_ are based on TFA measurements, while the κ_tot_est_/κ_lat_est_ are calculated based on the thermal conductivity of bulk CZTS/Ag taken from Sharma et al.^27^ (b) The estimated temperature-dependent *zT* values for the 24 h annealed CZFCTS-1 thin film. The uncertainties in *zT* are estimated to be ±20% based on the uncertainties in the other parameters in eq 1 and are comparable with values reported in earlier investigations.^67,68^

With the exception of the 24 h annealed thin films, all the films deposited on the TFA test chip contained too many structural defects for reliable thermal conductivity measurements. Moreover, the presence of small cracks in the 24 h annealed thin film deposited on the TFA test chip (Figure S15a) meant that the κ_tot_ and therefore the κ_lat_ determined this way are likely to be underestimated, which we denote as κ_tot_TFA_ and κ_lat_TFA_ respectively. We also estimate thermal conductivity by taking the measured κ_lat_ of bulk CZTS/Ag samples with a similar grain size to that of our thin films^27^ and combining this with the κ_ele_ obtained from the Wiedemann–Franz Law. We denote these values κ_lat_est_ and κ_tot_est_. As shown in Figure 7a, the κ_ele_ of the 24 h annealed thin film increases with temperature, mirroring the increase in σ (c.f. Figure 6b). Based on the thermal conductivities obtained from the experimental data and the κ_tot_ of bulk CZTS/Ag (vide supra), we estimate the κ_tot_ for CZFCTS-1 thin films to range between 0.17 and 0.68 W m^–1^ K^–1^ at 330–550 K.

By using the measured and estimated κ_tot_ values and combining these with the charge transport data in Figure 6, the *zT* values for the 24 h annealed CZFCTS-1 thin film were calculated as a function of temperature (Figure 7b), yielding an estimated peak *zT* of 0.18–0.69 at 550 K. We recognize the limitations of this approach and note that the upper bound for *zT* could be overestimated. However, it does provide the first estimates of *zT* for CZFCTS thin films. To improve the accuracy of TFA measurements of CZFCTS thin films, and subsequent *zT* evaluation, other deposition techniques could be explored for the preparation of high-quality, stress-free thin films on TFA test chips. To further optimize thermoelectric properties, it would be worth investigating the effects of different doping ratios, deposition temperatures and possibly postannealing under different conditions such as a sulfidation atmosphere.

## 4 Conclusions

In summary, high-quality single-phase CZTS and CZFCTS thin films were successfully deposited on glass substrates using AACVD; the atomic ratios could easily be controlled by adjusting the concentrations of the corresponding precursors. Cosubstitution of high levels of Fe and Cd into the CZTS lattice was found to increase the degree of structural distortion and to significantly improve the electrical transport properties. As-deposited single-phase CZFCTS thin films exhibited maximum PF values of ∼0.22 μW cm^–1^ K^–2^ at 575 K. Postdeposition annealing led to an improvement in the microstructure and enhanced thermoelectric performance. CZFCTS thin films annealed at 390 °C for 24 h showed a significantly improved maximum PF of ∼2.4 μW cm^–1^ K^–2^; this is higher than all reported values for single-phase chalcopyrite-like quaternary sulfide thin films and even exceeds most similar polycrystalline quaternary sulfide bulk materials to date. The thermal conductivity and *zT* values for the best CZFCTS thin films obtained after 24 h of postdeposition annealing were evaluated from a combination of TFA measurements and published data, and the maximum *zT* was estimated to be 0.18–0.69 at 550 K. This is the first reported *zT* evaluation for CZTS-based thin films. This study demonstrates an effective route to synthesize high-quality CZTS-based thin films with exceptional thermoelectric performance using AACVD, atomic substitution, and postdeposition annealing. Such CZFCTS thin films with the favorable thermoelectric properties presented in this work have the potential for thermoelectric applications and could be employed, for example, as the p-type leg of small-scale thermoelectric devices.

## Data Availability

All research data supporting this work are directly available within this publication.

## Supporting Information Available

Crystal structures of CZTS; synthesis of precursors; thermal decomposition of precursors; in situ variable temperature XRD patterns; effect of precursor ratios on film composition; HAADF-STEM analysis and EDX elemental maps for the CZFCTS-1 thin film; Raman, UV–vis absorption, and X-ray photoelectron spectroscopies; effect of postdeposition annealing; repeat measurement of electrical transport properties of the CZFCTS-1 thin film annealed for 24 h; estimation of the thermal conductivity and *zT* (PDF)
