# Supplementary material for: Exceptional Thermoelectric Performance of Cu2(Zn,Fe,Cd)SnS4 Thin Films
Source: ACS Appl Mater Interfaces. 2024 Feb 23;16(9):11516–27. doi: 10.1021/acsami.3c17730 (PMC10921374; doi:10.1021/acsami.3c17730)
Supplement: Supplementary file 1 — am3c17730_si_001.pdf [file am3c17730_si_001.pdf]

Supporting Information

Exceptional Thermoelectric Performance of $Cu_2(Zn,Fe,Cd)SnS_4$ Thin Films

Yu Liu,[a] Paul D. McNaughter,[b] Xiaodong Liu,[a] Andrey V. Kretinin,[a, c] Jonathan M. Skelton,[b] Feridoon Azough,[a] David J. Lewis,* [a] and Robert Freer* [a]

[a] *Department of Materials, University of Manchester, Oxford Road, Manchester, M13 9PL, UK.*

[b] *Department of Chemistry, University of Manchester, Oxford Road, Manchester, M13 9PL, UK.*

[c] *National Graphene Institute, University of Manchester, Oxford Road, Manchester, M13 9PL, UK.*

*Corresponding authors: Robert Freer, David J. Lewis; e-mail: Robert.Freer@manchester.ac.uk and david.lewis-4@manchester.ac.uk

Crystal Structures of CZTS

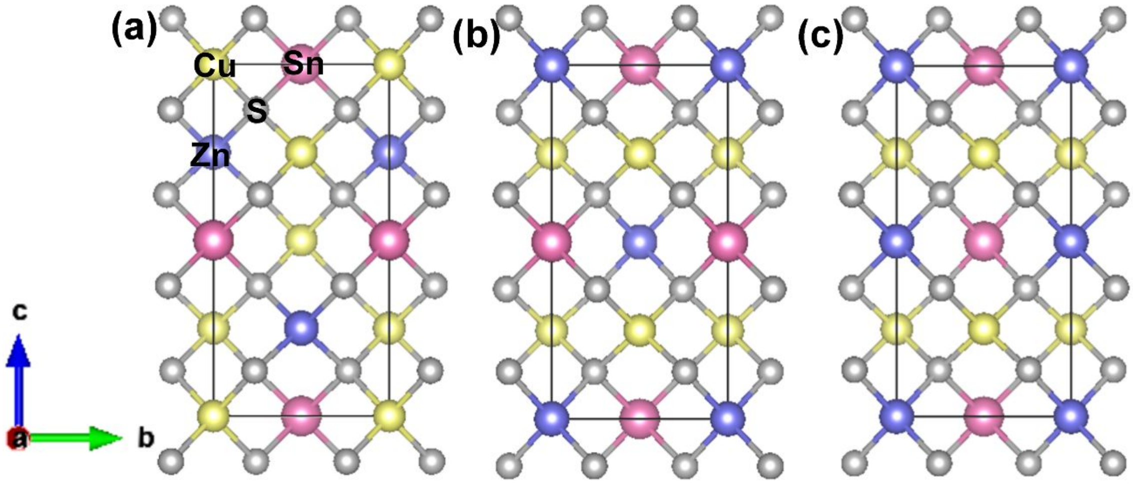

Figure S1. Crystal structures of the kesterite (a), stannite (b) and PMCA (c) structures of Cu_2ZnSnS_4 (CZTS).

Synthesis of Precursors

The synthesis of the diethyldithiocarbamate precursors was conducted using a standard Schlenk system under an inert atmosphere of dry nitrogen. All reagents were purchased from Sigma-Aldrich and used as received. Elemental analysis was conducted by the micro-analytical laboratory at The University of Manchester.

Synthesis of bis(diethyldithiocarbamate)copper(II) [$Cu(S_2CN(C_2H_5)_2)_2$]: $Na(S_2CN(C_2H_5)_2)\cdot 3H_2O$ (0.04 mol, 9.0 g) and $CuCl_2\cdot 2H_2O$ (0.02 mol, 3.4 g) were separately dissolved in methanol and the $CuCl_2$ solution added dropwise to the $Na(S_2CN(C_2H_5)_2)$ solution and stirred for 1 h. The resulting black precipitate was isolated by vacuum filtration, washed using 100 mL methanol and dried under vacuum at room temperature. Yield: 5.2 g (72%). Elemental analysis: calculated values for $C_{10}H_{20}N_2S_4Cu$ - C, 33.36%; H, 5.60%; N, 7.78%; S, 35.62%; Cu, 17.65%; found - C, 33.28%; H, 5.62%; N, 7.69%; S, 35.64%; Cu, 17. 05%.

Synthesis of bis(diethyldithiocarbamate)zinc(II) [$Zn(S_2CN(C_2H_5)_2)_2$]: $Zn(S_2CN(C_2H_5)_2)_2$ was prepared using a similar procedure to that outlined above. $Na(S_2CN(C_2H_5)_2)\cdot 3H_2O$ (0.04 mol, 9.0 g) was dissolved in 200 mL methanol and stirred for 30 min. $Zn(CH_3COO)_2$ (0.02 mol, 3.6 g) was mixed with methanol and the resulting $Zn(CH_3COO)_2$ suspension was added dropwise to the

$Na(S_2CN(C_2H_5)_2)$ solution and stirred for 1 h. The resulting white precipitate was isolated by vacuum filtration, washed using 100 mL methanol and dried under vacuum at room temperature. Yield: 7.0 g (95%). Elemental analysis: calculated values for $C_{10}H_{20}N_2S_4Zn$ - C, 33.19%; H, 5.57%; N, 7.74%; S, 35.44%; Zn, 18.07%; found - C, 33.15%; H, 5.56%; N, 7.64%; S, 35.15%; Zn, 17. 78%.

Synthesis of bis(diethyldithiocarbamate)iron(III) [$Fe(S_2CN(C_2H_5)_2)_3$]: A similar method to that described above was used to synthesize $Fe(S_2CN(C_2H_5)_2)_3$. $Na(S_2CN(C_2H_5)_2)\cdot 3H_2O$ (0.06 mol, 13.5 g) and $FeCl_3$ (0.02 mol, 3.2 g) were separately dissolved in methanol and the $FeCl_3$ solution was added dropwise to the $Na(S_2CN(C_2H_5)_2)$ solution and stirred for 1 h. The resulting black precipitate was isolated by vacuum filtration, washed using 100 mL methanol and dried under vacuum at room temperature. Yield: 9.07 g (90%). Elemental analysis: calculated values for $C_{15}H_{30}N_3S_6Fe$ - C, 35.99%; H, 6.04%; N, 8.39%; S, 38.43%; Fe, 11.15%; found - C, 35.91%; H, 6.00%; N, 8.32%; S, 38.60%; Fe, 11.29%.

Synthesis of bis(diethyldithiocarbamate)cadmium(II) [$Cd(S_2CN(C_2H_5)_2)_2$]: $Cd(S_2CN(C_2H_5)_2)_2$ was prepared using a similar procedure to that described above. $Na(S_2CN(C_2H_5)_2)\cdot 3H_2O$ (0.04 mol, 9.0 g) was dissolved in 200 mL methanol and stirred for 30 min. $CdCl_2$ (0.02 mol, 3.6 g) was mixed with methanol and the $CdCl_2$ suspension was added dropwise to the $Na(S_2CN(C_2H_5)_2)$ solution and stirred for 1 h. The resulting white precipitate was isolated by vacuum filtration, washed using 100 mL methanol and dried under vacuum at room temperature. Yield: 6.6 g (81%). Elemental analysis: calculated values for $C_{10}H_{20}N_2S_4Cd$ - C, 29.37%; H, 4.93%; N, 6.85%; S, 31.36%; Cd, 27.49%; found - C, 28.95%; H, 4.67%; N, 6.44%; S, 30.91%; Cd, 27.15%.

Synthesis of dibutyl-bis(diethyldithiocarbamate)tin(IV) [$Sn(C_4H_9)_2(S_2CN(C_2H_5)_2)_2$]: $Na(S_2CN(C_2H_5)_2)\cdot 3H_2O$ (0.04 mol, 9.0 g) and Bu_2SnCl_2 (0.02 mol, 6.1 g) were separately dissolved in ethanol and the Bu_2SnCl_2 solution was added dropwise to the $Na(S_2CN(C_2H_5)_2)$ solution and stirred for 1 h. The white NaCl precipitate was removed by vacuum filtration and the volume of the filtrate was reduced to ~50 mL using vacuum distillation. The resulting pale yellow solution was then cooled to ~4 ℃ for recrystallization, yielding colourless crystals that were separated by filtration, washed with 100 mL cool ethanol, and dried under vacuum at room temperature. Yield: 8.8 g (83%). Elemental analysis: calculated values for $C_{18}H_{38}SnN_2S_4$ - C, 40.83%; H, 7.23%; N, 5.29%; S, 24.22%; Sn, 22.42%; found - C, 40.49%, H 7.29%, N 5.20%, S 23.62%, Sn 22.47%.

Thermal Decomposition of Precursors

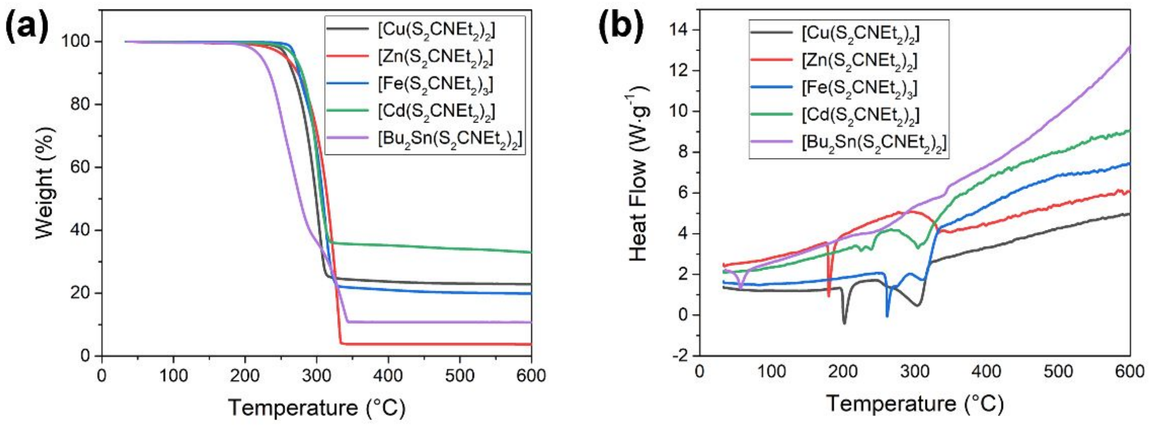

Figure S2. (a) TGA and (b) DSC analysis of $[Cu(S_2CN(C_2H_5)_2)_2]$, $[Zn(S_2CN(C_2H_5)_2)_2]$, $[Fe(S_2CN(C_2H_5)_2)_3]$, $[Cd(S_2CN(C_2H_5)_2)_2]$, and $[Sn(C_4H_9)_2(S_2CN(C_2H_5)_2)_2]$.

Figure S2 shows the TGA and DSC traces measured for the five precursors. The TGA data indicates that the decomposition of $[Sn(C_4H_9)_2(S_2CN(C_2H_5)_2)_2]$ occurs between 200-350 ℃ with a residual mass of ~11% (expected value for SnS: ~28%). $[Zn(S_2CN(C_2H_5)_2)_2]$ decomposes between 240-330 ℃ with a residual mass of ~4% (expected value for ZnS: ~27%). $[Cu(S_2CN(C_2H_5)_2)_2]$ decomposes between 250-320 ℃ with a residual mass of ~23% (expected value for CuS: ~27%). $[Cd(S_2CN(C_2H_5)_2)_2]$ decomposes between 260-310 ℃ with a residual mass of ~32% (expected value for CdS: ~35%). $[Fe(S_2CN(C_2H_5)_2)_3]$ decomposes between 260-325 ℃ with a residual mass of ~20% (expected value for FeS: ~25%). The measured residual masses of the precursors after decomposition are all lower than the calculated values due to sublimation, as observed in previous work.[1]

The DSC data show obvious endothermic peaks for the decompositions of all precursors. The sharper endothermic peaks might result from the melting of the precursors, whilst the broader endothermic peaks possibly result from the decomposition. After these endothermic events, heat flow increases steadily for all the precursors, indicating continued crystallization.[2] The TGA and DSC characterisation of the precursors is consistent with published data.[3-4]

In-situ Variable Temperature XRD Patterns

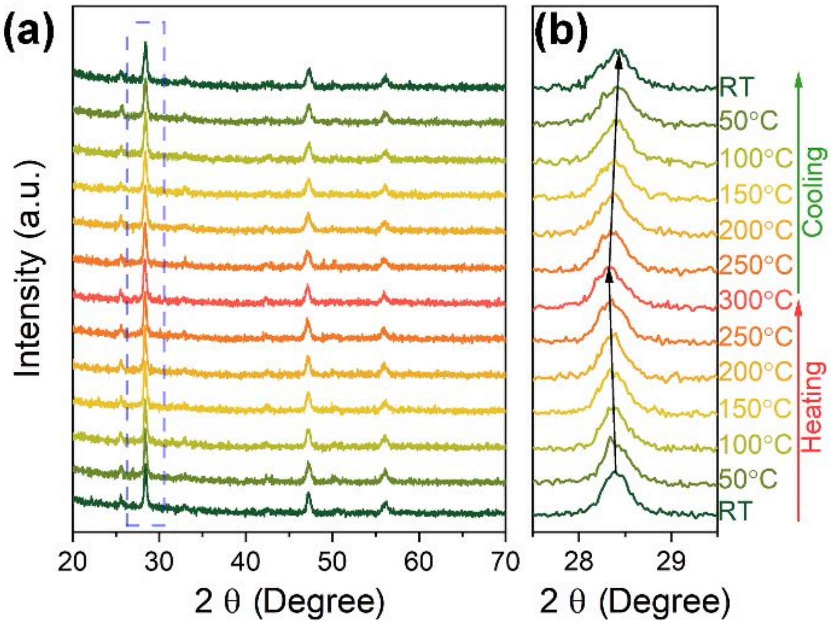

Figure S3. *In situ* XRD patterns for the CZFCTS-1 thin film from room temperature (RT) to 300 °C. Subplot (b) shows an expansion of the region marked by the blue box in (a).

Effect of Precursor Ratios on Film Composition

Figures S4a and S4b show the stoichiometries of the prepared CZTS and CZFCTS thin films. From Figure S4a, it is clear the stoichiometries of the CZTS thin films are close to the theoretical values, indicating that close-to-stoichiometric CZTS thin films can be prepared by AACVD. In contrast, the CZFCTS thin films have similar ratios of Cu:Sn:B-site (Figure S4b), with differences below 6%, but show Sn sufficiency and Cu deficiency compared to the CZTS thin films. Figure S4b shows the Fe/B-site, Zn/B-site and Cd/B-site ratios in the CZFCTS thin films. The CZFCTS-1 thin films prepared with equimolar ratios of the Cd, Fe and Zn precursors show a larger Fe/B-site ratio than Cd/B-site and Zn/B-site ratios, possibly as a result of the higher solubility of Fe in the CZTS lattice.[5-6] Compared with the CZFCTS-1 thin film, the CZFCTS-2 thin film prepared with a lower concentration of the Fe precursor exhibits obviously reduced Fe/B-site ratio and increased Zn/B-site ratios. The CZFCTS-3 thin film, prepared with a 2:1:2 ratio of Zn:Fe:Cd and a higher total concentration of the B-site metal precursors, has a larger Fe/B-site ratio and smaller Cd/B-site and Zn/B-site ratios than the CZFCTS-2 thin film. Figure S4c shows the correlation between the mole fractions of the metal atoms in the precursor feed and those in the obtained thin films. The CZFCTS

thin films have a similar Cu percentage, whereas the CZFCTS-1 thin film exhibits the highest Fe content. Thus, the stoichiometry of CZFCTS thin films can be controlled to some degree by adjusting the ratios of the corresponding precursors in the feed.

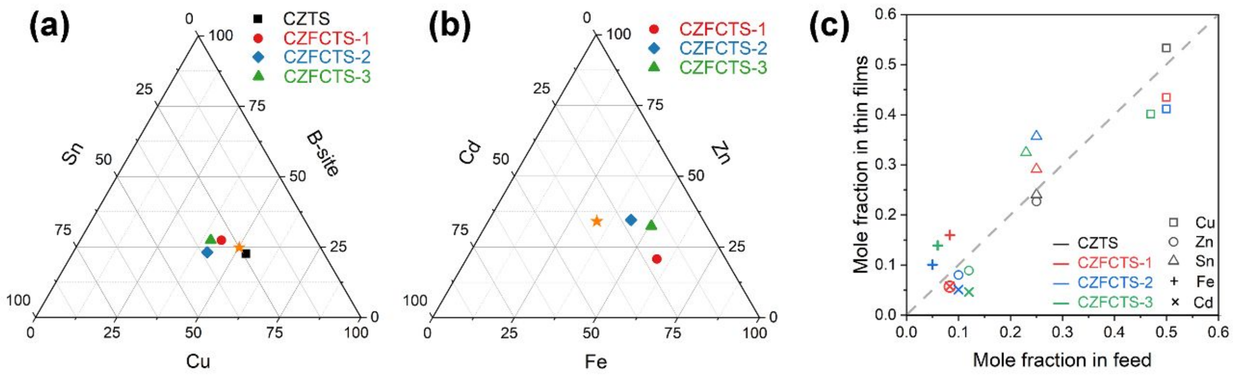

Figure S4. (a)/(b) Stoichiometries of the CZTS and CZFCTS thin films prepared using different precursor ratios (B-site = Zn + Fe + Cd). The orange stars in (a) and (b) represent stoichiometric Cu_2ZnSnS_4 and $Cu_2(Zn_{1/3}Fe_{1/3}Cd_{1/3})SnS_4$ respectively. (c) Relationship between the mole fractions of the metal atoms in the films and mole fractions of the precursors in the feed.

HAADF-STEM Analysis and EDX Elemental Maps for the CZFCTS-1 Thin Film

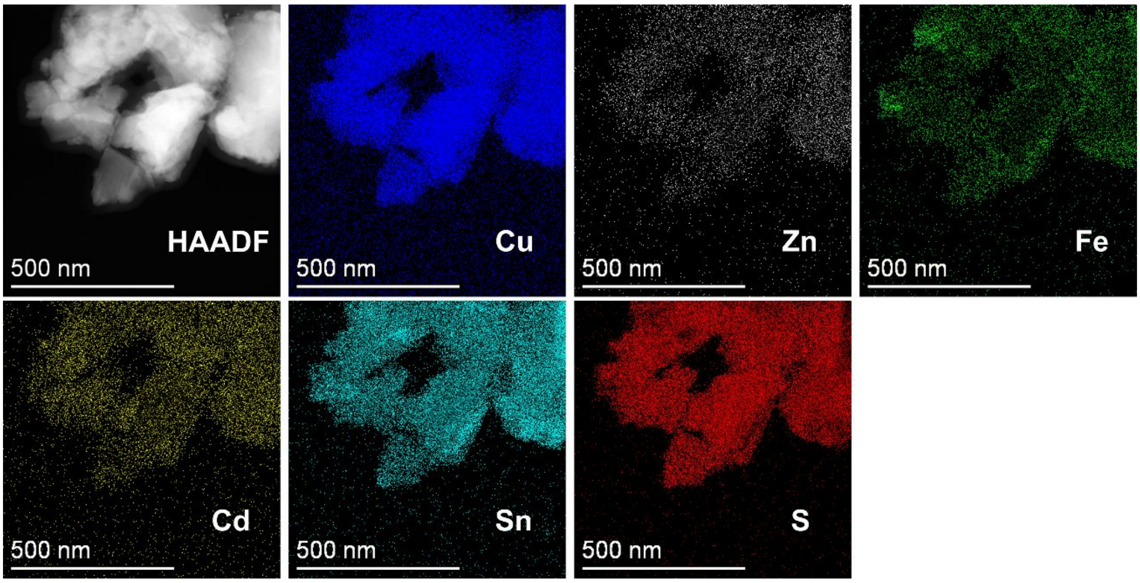

Figure S5. HAADF-STEM image and EDX elemental maps of Cu, Zn, Fe, Cd, Sn and S for the CZFCTS-1 thin film.

Rietveld Refinement

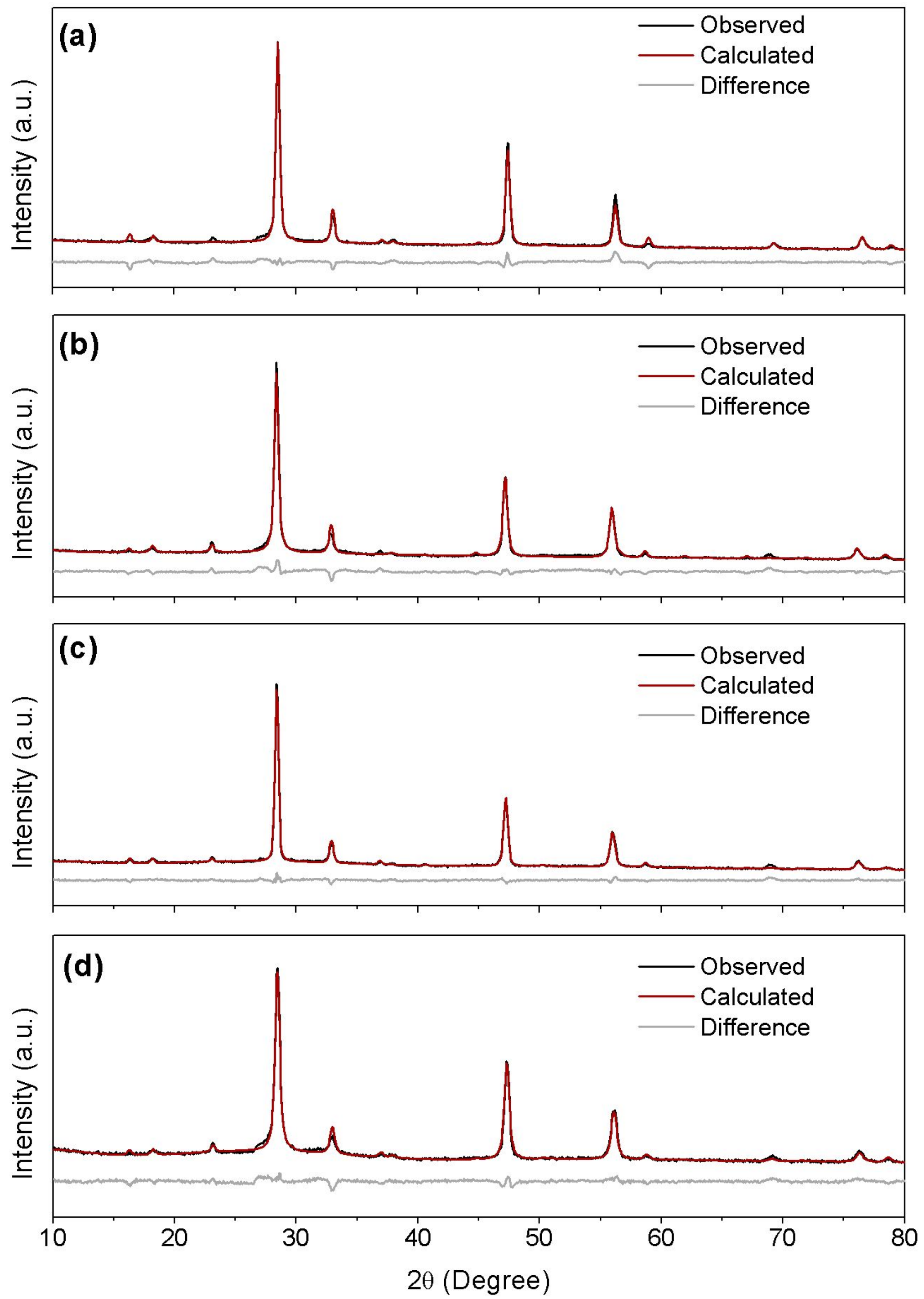

Figure S6. Rietveld refined XRD patterns for (a) CZTS, (b) CZFCTS-1, (c) CZFCTS-2, and (d) CZFCTS-3 thin films. [Instrument alignment and correction settings during Rietveld refinement – Instrument: Primary radius (mm) 200; Secondary radius (mm) 200; Receiving slit width (mm) 0.1.

Full Axial Convolution: Filament length (mm) 10; Sample length (mm) 15; Receiving Slit length (mm) 12; Primary Sollers (°) 2.3; Secondary Sollers (°) 2.3. Correction: LP Factor 26.4].

Raman and UV-VIS Analysis

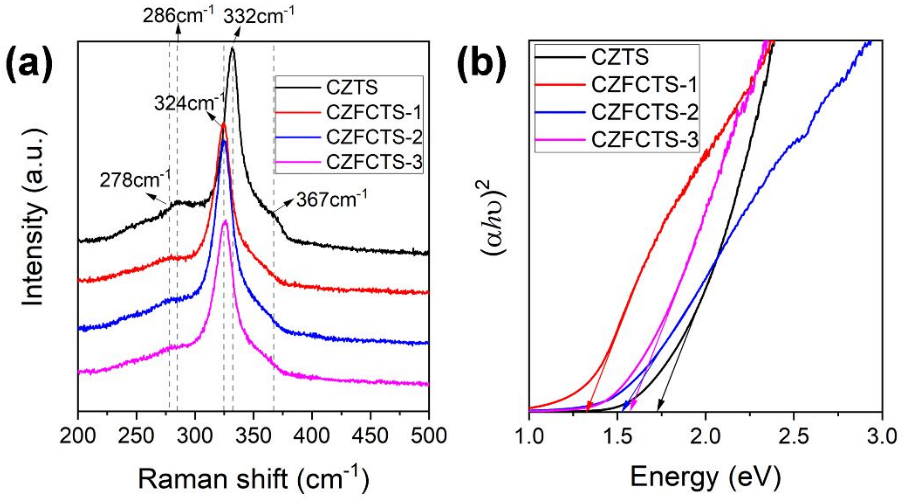

Figure S7. (a) Raman spectra and (b) optical bandgap measurements for the CZTS and CZFCTS thin films prepared using different precursor ratios.

The optical band gap energies of the CZTS and CZFCTS films were estimated from the absorption spectra using the relation $(\alpha hv)^n = A(hv - E_g)$,[7] where α is the absorption coefficient, hv is photon energy, A is a constant, and E_g is the bandgap. In the present work, the equation is satisfied for n = 2, implying direct allowed transitions in both the CZTS and CZFCTS thin films. The bandgap of the CZTS thin film is ~1.7 eV, in good agreement with other reported values for CZTS thin films.[8-9] In comparison, the bandgaps of the CZFCTS thin films range from ~1.3-1.6 eV and are smaller than those of the CZTS thin film, consistent with previous work.[10-11] The CZFCTS-1 thin film, which has higher Fe content, exhibits the the smallest bandgap of ~1.3 eV.

XPS Analysis

Table S1. Binding energies determined from XPS measurements on the CZTS and CZFCTS thin films.

		CZTS		CZFCTS	
Element	Energy level	Binding energy (eV)	Peak separation (eV)	Binding energy (eV)	Peak separation (eV)
Cu(I)	2p $_{1/2}$	952.1	19.9	951.6	19.9
	2p $_{3/2}$	932.2		931.7	
Zn(II)	2p $_{1/2}$	1045.2	23.1	1045.1	23.1
	2p $_{3/2}$	1022.1		1022.0	
Sn(IV)	3d $_{3/2}$	495.3	8.5	494.9	8.4
	3d $_{5/2}$	486.8		486.5	
S(VI)	2p $_{1/2}$	162.8	1.1	162.4	1.2
	2p $_{3/2}$	161.7		161.2	
Fe(II)	2p $_{1/2}$	------	------	724.5	13.5
	2p $_{3/2}$	------		711.0	
Cd(II)	3d $_{3/2}$	------	------	411.6	6.7
	3d $_{5/2}$	------		404.9	

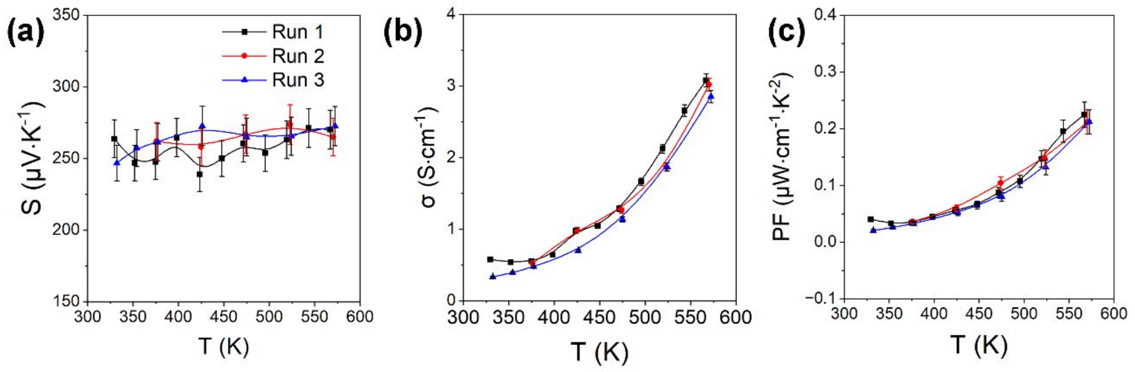

Figure S8. Repeat measurements of the temperature dependence of (a) the Seebeck coefficient (*S*), (b) electrical conductivity (σ) and (c) power factor (*PF*) of the CZFCTS-1 thin film. There is neglectable differences between the results obtained from repeat measurements, confirming the stability of the samples at the temperature of < 300°C.

Effect of Post-deposition Annealing

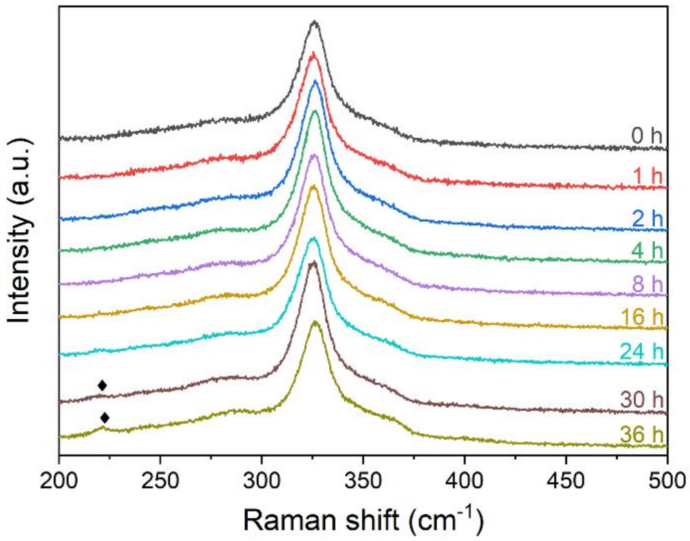

Figure S9. Raman spectra of unannealed CZFCTS-1 thin films (0 h) and films annealed for up to 36 h. The diamond symbols denote features attributed to FeS.

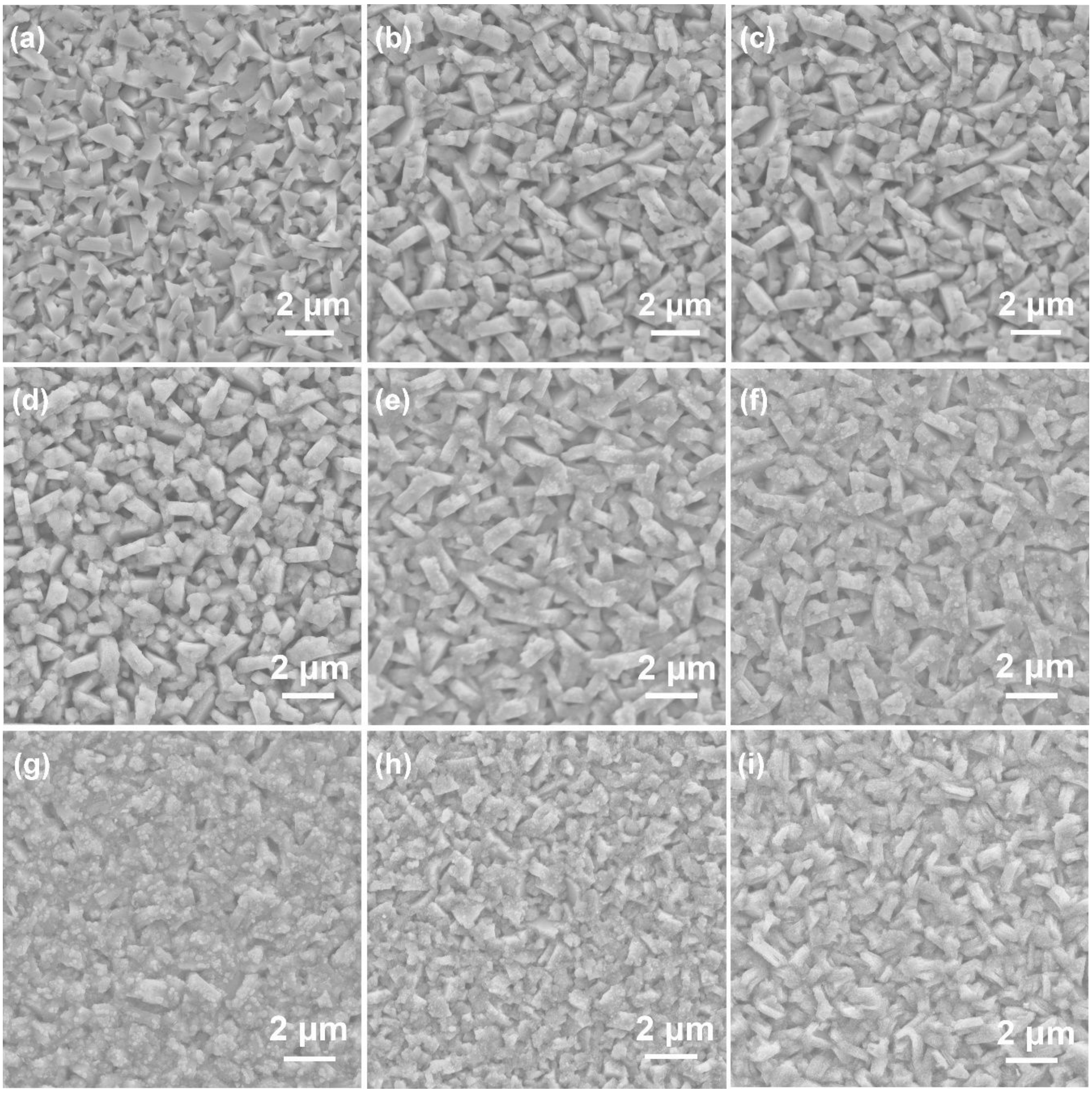

Figure S10. SEM images of CZFCTS-1 thin films annealed at 390℃ for (a) 0 h, (b) 1 h, (c) 2 h, (d) 4 h, (e) 8 h, (f) 16 h, (g) 24 h, (h) 30 h, and (i) 36 h.

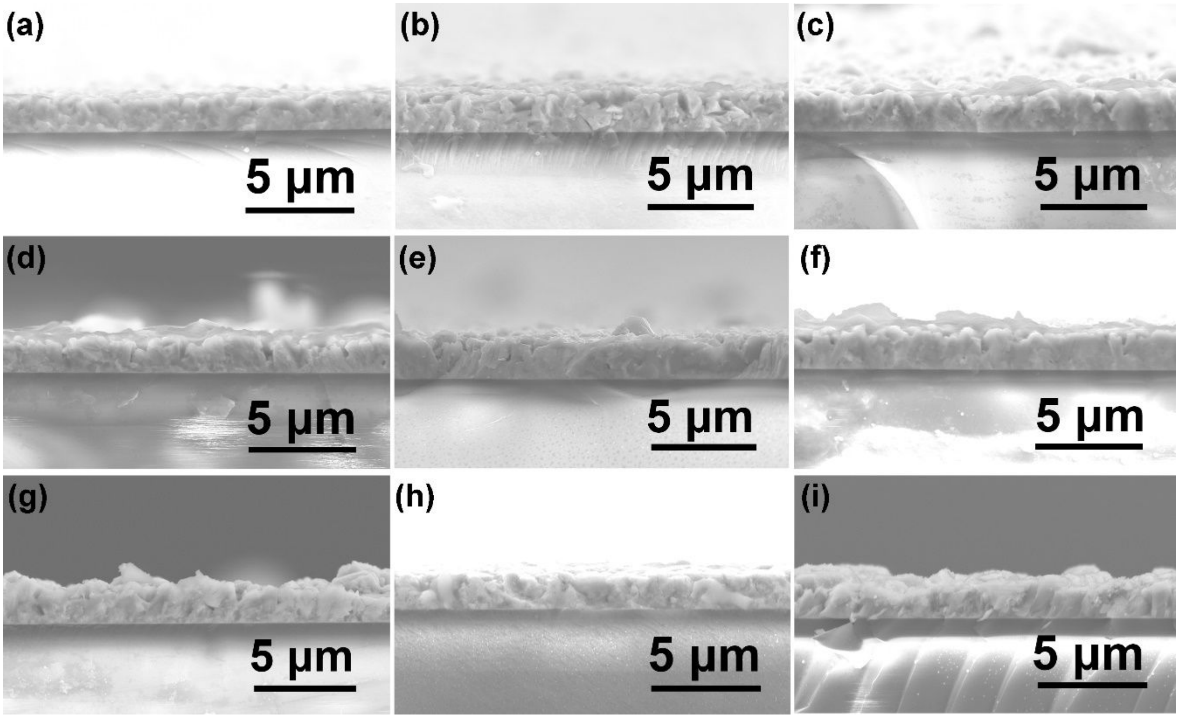

Figure S11. Cross-sectional SEM images for CZFCTS-1 thin films annealed at 390 ℃ for (a) 0 h, (b) 1 h, (c) 2 h, (d) 4 h, (e) 8 h, (f) 16 h, (g) 24 h, (h) 30 h, and (i) 36 h.

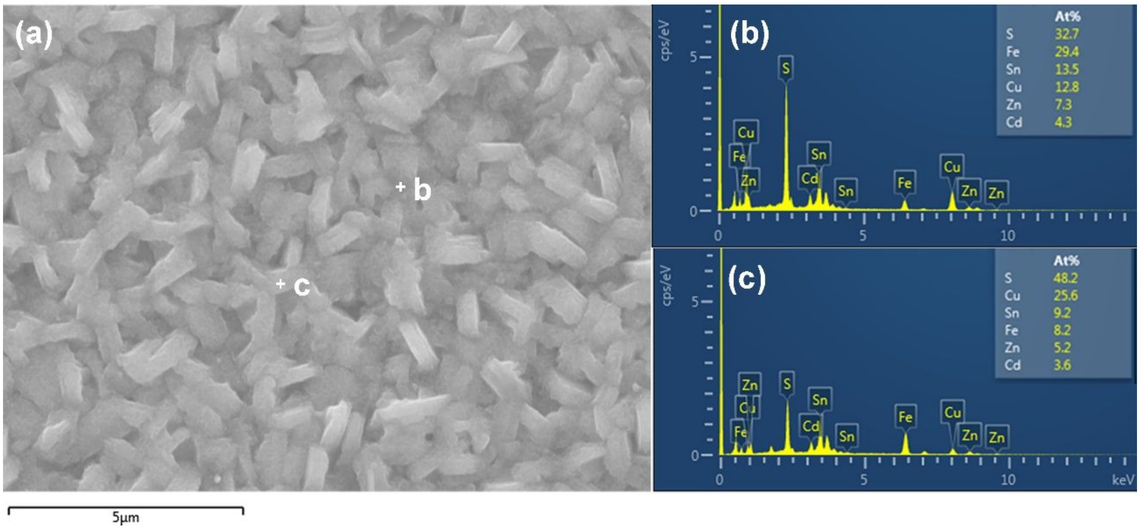

Figure S12. SEM image (a) and EDX elemental mapping (b), performed at the locations marked b and c in (a), for CZFCTS-1 thin films annealed for 36 h.

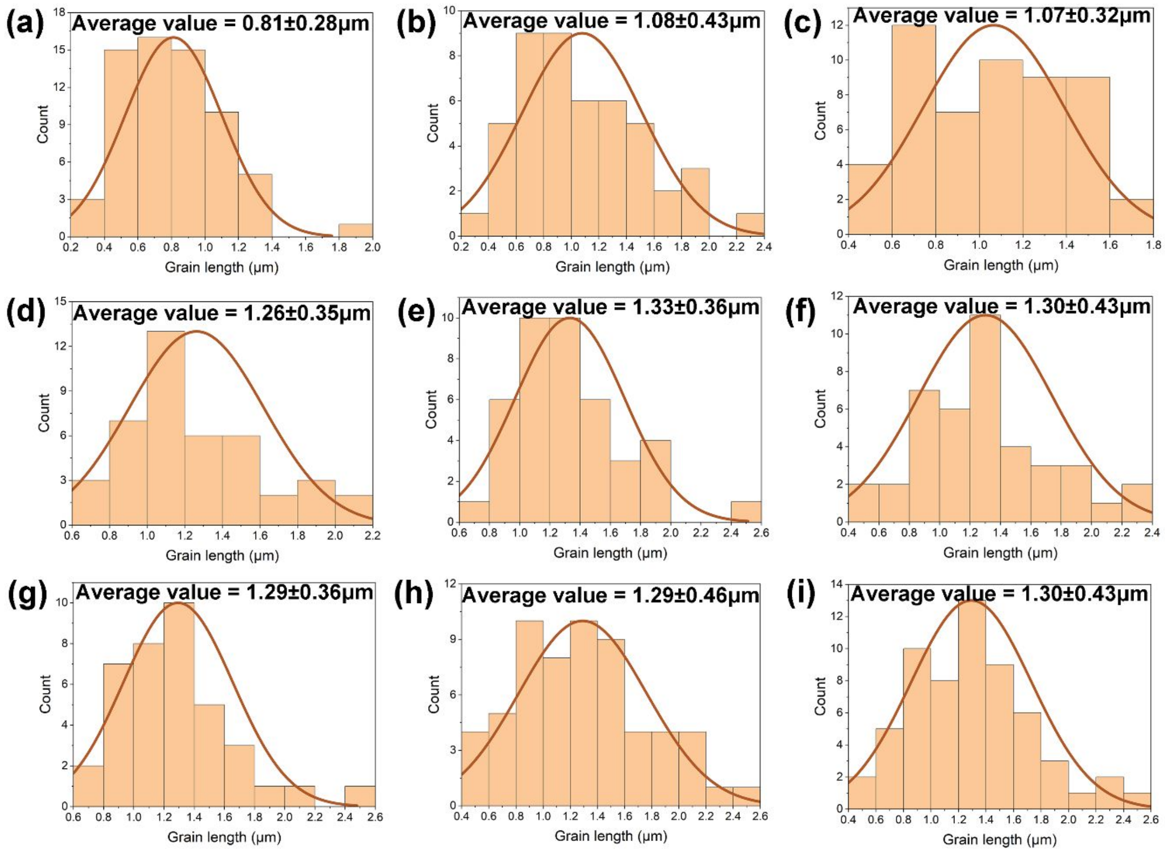

Figure S13. Average grain sizes and grain size distributions and for CZFCTS-1 thin films annealed at 390 ℃ for (a) 0 h, (b) 1 h, (c) 2 h, (d) 4 h, (e) 8 h, (f) 16 h, (g) 24 h, (h) 30 h, and (i) 36 h. The uncertainties in the average grain size are given as the standard deviations about the mean value.

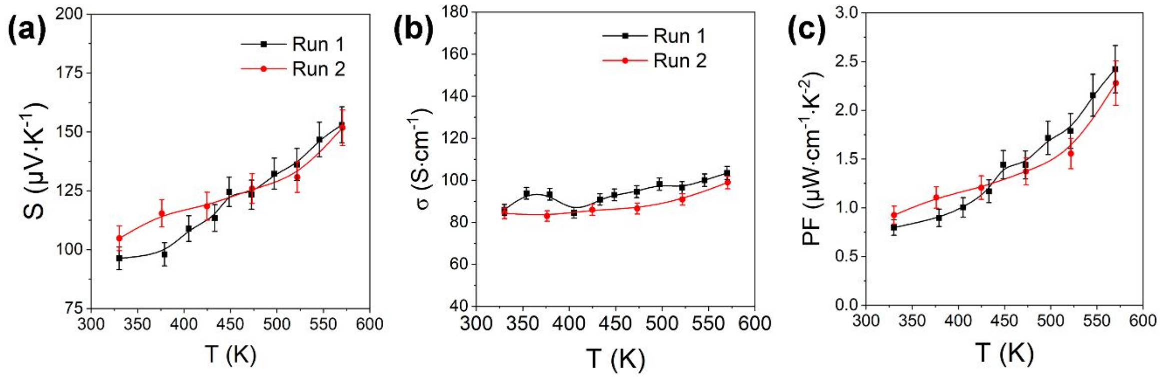

Figure S14. Repeat measurements of the temperature dependence of (a) the Seebeck coefficient (S), (b) electrical conductivity (σ) and (c) power factor (PF) of a CZFCTS-1 thin film annealed at 390 ℃ for 24 h.

Estimation of Thermal Conductivity and zT

As described in the text, the electronic thermal conductivity κ_{ele} was estimated from the electrical transport measurements and combined with the total thermal conductivity κ_{tot_TFA} measured using TFA to estimate κ_{lat_TFA}.

The morphology of the thin film deposited on the TFA test chip (Figure S15a) was slightly different from the films deposited on glass and contains small cracks, probably as a result of thermal stresses caused by the large difference in thermal expansion coefficients between the film and the Si_3N_4 membrane on the test chip. These cracks could lead to underestimation of the thermal conductivity.

We used the κ_{lat_est} reported for bulk CZTS/1 wt% Ag samples with a similar grain size to our thin films[12] to calculate a κ_{tot_est} from our measured κ_{ele}.

The estimated zT values were calculated from κ_{tot_TFA} and κ_{tot_est} from the relationship $zT = S^2\sigma T/\kappa_{tot}$. The uncertainty was estimated to be 20% based on uncertainties in the other parameters in equation (1); this is comparable with values reported in earlier investigations.[13-14]

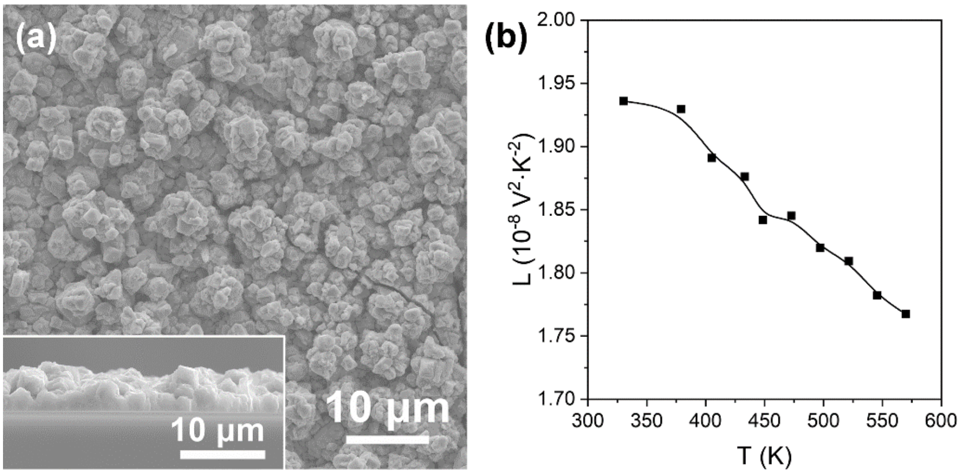

Figure S15. (a) SEM and cross-sectional images of the CZFCTS-1 thin film deposited on the TFA test chip. (b) Temperature dependence of the calculated Lorenz number L for the CZFCTS-1 thin film annealed for 24 h.

References

(1) Kevin, P.; Malik, M. A.; O'Brien, P., The Controlled Deposition of $Cu_2(Zn_yFe_{1-y})SnS_4$, $Cu_2(Zn_yFe_{1-y})SnSe_4$ and $Cu_2(Zn_yFe_{1-y})Sn(S_xSe_{1-x})_4$ Thin Films by AACVD: Potential Solar Cell Materials Based on Earth Abundant Elements. *J. Mater. Chem. C* **2015,** *3*, 5733-5741.

(2) Sabu, U.; Tripathi, N.; Logesh, G.; Rashad, M.; Joy, A.; Balasubramanian, M., Development of Biomorphic C-ZnO with In-situ Formation of ZnS Using Eggshell Membrane as Bio-template. *Ceram. Int.* **2020,** *46*, 22869-22875.

(3) Kevin, P.; Malik, M. A.; O'Brien, P., The AACVD of $Cu_2FeSn(S_xSe_{1-x})_4$: Potential Environmentally Benign Solar Cell Materials. *New J. Chem.* **2015,** *39*, 7046-7053.

(4) Murtaza, G.; Alderhami, S.; Alharbi, Y. T.; Zulfiqar, U.; Hossin, M.; Alanazi, A. M.; Almanqur, L.; Onche, E. U.; Venkateswaran, S. P.; Lewis, D. J., Scalable and Universal Route for the Deposition of Binary, Ternary, and Quaternary Metal Sulfide Materials from Molecular Precursors. *ACS Appl. Energy Mater.* **2020,** *3*, 1952-1961.

(5) Huang, K. L.; Huang, C. H.; Lin, W. T.; Fu, Y. S.; Guo, T. F., Solvothermal Synthesis and Tunable Bandgap of $Cu_2(Zn_{1-x}Co_x)SnS_4$ and $Cu_2(Fe_{1-x}Co_x)SnS_4$ Nanocrystals. *J. Alloys Compd.* **2015,** *646*, 1015-1022.

(6) Kevin, P.; Malik, M. A.; McAdams, S.; O'Brien, P., Synthesis of Nanoparticulate Alloys of the Composition $Cu_2Zn_{1-x}Fe_xSnS_4$: Structural, Optical, and Magnetic Properties. *J. Am. Chem. Soc.* **2015,** *137*, 15086-15089.

(7) Gurubhaskar, M.; Thota, N.; Raghavender, M.; Hema Chandra, G.; Prathap, P.; Venkata Subbaiah, Y. P., Influence of Sulfurization Time on Two Step Grown SnS Thin Films. *Vacuum* **2018,** *155*, 318-324.

(8) Pandiyan, R.; Oulad Elhmaidi, Z.; Sekkat, Z.; Abd-lefdil, M.; El Khakani, M. A., Reconstructing the Energy Band Electronic Structure of Pulsed Laser Deposited CZTS Thin Films Intended for Solar Cell Absorber Applications. *Appl. Surf. Sci.* **2017,** *396*, 1562-1570.

(9) Hunge, Y. M.; Mahadik, M. A.; Patil, V. L.; Pawar, A. R.; Gadakh, S. R.; Moholkar, A. V.; Patil, P. S.; Bhosale, C. H., Visible Light Assisted Photoelectrocatalytic Degradation of Sugarcane Factory Wastewater by Sprayed CZTS Thin Films. *J. Phys. Chem. Solids* **2017,** *111*, 176-181.

(10) Agawane, G. L.; Shin, S. W.; Vanalakar, S. A.; Moholkar, A. V.; Kim, J. H., Next Generation Promising $Cu_2(Zn_xFe_{1-x})SnS_4$ Photovoltaic Absorber Material Prepared by Pulsed Laser Deposition Technique. *Mater. Lett.* **2014,** *137*, 147-149.

(11) Su, Z.; Tan, J. M. R.; Li, X.; Zeng, X.; Batabyal, S. K.; Wong, L. H., Cation Substitution of Solution-Processed Cu_2ZnSnS_4Thin Film Solar Cell with over 9% Efficiency. *Adv. Energy Mater.* **2015,** *5*, 1500682.

(12) Sharma, S. D.; Khasimsaheb, B.; Chen, Y. Y.; Neeleshwar, S., Enhanced Thermoelectric Performance of Cu_2ZnSnS_4 (CZTS) by Incorporating Ag Nanoparticles. *Ceram. Int.* **2019,** *45*, 2060-2068.

(13) Zhao, L.-D.; Lo, S.-H.; Zhang, Y.; Sun, H.; Tan, G.; Uher, C.; Wolverton, C.; Dravid, V. P.; Kanatzidis, M. G., Ultralow Thermal Conductivity and High Thermoelectric Figure of Merit in SnSe Crystals. *Nature* **2014,** *508*, 373-377.

(14) Wang, H.; Pei, Y.; LaLonde, A. D.; Snyder, G. J., Heavily Doped P-type PbSe with High Thermoelectric Performance: An Alternative for PbTe. *Adv. Mater.* **2011,** *23*, 1366-1370.
